# Spatial and single-cell insights into the prostate cancer microenvironment: overcoming immunotherapy resistance through multidimensional strategies

**DOI:** 10.3389/fimmu.2026.1979117

**Published:** 2026-10-22

**Authors:** Dekai Liu, Junliang Tang, Yiwei Jiang, Jungang Zhao, Junxiao Shen, Lijie You, Yichun Zheng

**Affiliations:** 1 Department of Urology, the Fourth Affiliated Hospital of School of Medicine, and International School of Medicine, International Institutes of Medicine, Zhejiang University, Yiwu, China; 2 Department of Urology, the Second Affiliated Hospital of School of Medicine, Zhejiang University, Hangzhou, China

**Keywords:** immunosuppression, immunotherapy resistance, prostate cancer, single-cell RNA sequencing, spatial transcriptomics, tumor microenvironment

## Abstract

Despite breakthroughs in immune checkpoint inhibitors (ICIs) across various malignancies, prostate cancer (PCa), particularly metastatic castration-resistant prostate cancer (mCRPC), has shown limited responses to ICIs. This pervasive primary resistance is fundamentally driven by the canonically “immune-cold” and highly immunosuppressive tumor microenvironment (TME) of PCa. Propelled by recent advancements in single-cell RNA sequencing and spatial transcriptomics, this review comprehensively deconstructs the dynamic spatiotemporal evolution of the PCa immune landscape across localized, mCRPC, and lineage-plastic variant stages (e.g., neuroendocrine and double-negative PCa). We systematically delineate how the profound immune-excluded phenotype is sculpted not by isolated events, but by an intricate, multicellular suppressive ecosystem. Specifically, we detail the subclonal heterogeneity, spatial topography, and metabolic crosstalk of key suppressive populations, including specific tumor-associated macrophage (TAM) subsets, regulatory T cells (Tregs), cancer-associated fibroblasts (CAFs), and myeloid-derived suppressor cells (MDSCs). Furthermore, we elucidate the tumor-intrinsic genetic aberrations (e.g., PTEN inactivation, TP53 mutations) and epigenetic rewiring (e.g., EZH2, CHD1, ARID1A dysregulation) that initiate and perpetually orchestrate this suppressive network. Finally, we highlight emerging therapeutic vulnerabilities and future translational directions. Dismantling the microenvironmental barriers in PCa necessitates transcending singular ICI blockade by employing novel combinatorial strategies that target chemotactic recruitment axes (e.g., CXCR2/CXCR4), reverse metabolic plasticity, and abrogate epigenetic silencing. Coupled with high-resolution liquid biopsy biomarkers (e.g., ctDNA, B7-H3+ EVs) for precise patient stratification and cutting-edge synthetic biology interventions, these multidimensional approaches hold profound promise for reversing the dual metabolic and immune suppressive state of PCa, thereby overcoming clinical immunotherapy resistance.

## 1 Introduction

Prostate cancer (PCa) represents one of the most prevalent malignancies among men globally, imposing a profound public health burden. According to the 2025 annual cancer statistics released by the American Cancer Society (ACS), the incidence of PCa has steadily climbed by approximately 3% annually since 2014, while its mortality rate remains notoriously prominent among male malignancies, reaching 0.019% (1). Despite the recent advent of novel chemotherapeutic agents, targeted interventions, and radioligand therapies, androgen deprivation therapy (ADT) remains the cornerstone of clinical management for advanced and metastatic PCa, primarily owing to its efficacy in extinguishing androgen receptor (AR) signaling—the central oncogenic driver of PCa proliferation (2). However, under the therapeutic pressure of ADT, an estimated 10% to 20% of patients inevitably progress to castration-resistant prostate cancer (CRPC). Crucially, 84% of this subset presents with concurrent bone metastases, culminating in metastatic CRPC (mCRPC) (3). Characterized by profound tumor heterogeneity and relentless clinical aggressiveness, mCRPC portends a dismal prognosis with significantly curtailed 5-year survival rates. Given that existing sequential therapies offer limited survival benefits, managing this aggressive phenotype remains a significant clinical challenge (4).

Although immune checkpoint inhibitors (ICIs)—exemplified by programmed cell death-1/its ligand (PD-1/PD-L1) and cytotoxic T-lymphocyte-associated protein-4 (CTLA-4) blockades—have yielded breakthrough clinical benefits across diverse solid tumors such as melanoma and non-small cell lung cancer, their objective response rates in the vast majority of prostate cancer patients, particularly in the mCRPC setting, remain notoriously low (5–7). This pervasive primary resistance to ICIs is fundamentally rooted in the canonically “immune-cold” phenotype of the prostate tumor microenvironment (TME) (8). Rather than being driven by a singular molecular event or an isolated cell lineage, this profoundly immune-excluded phenotype is actively sculpted by a highly heterogeneous and dynamically interacting ecosystem. On one hand, tumor-intrinsic genetic and epigenetic aberrations not only constrain neoantigen generation but also engage aberrant intracellular signaling cascades to actively suppress immune cell chemotaxis and infiltration (9–11). On the other hand, the TME relentlessly recruits and accumulates vast populations of potently immunosuppressive cells, most notably tumor-associated macrophages (TAMs), regulatory T cells (Tregs), and myeloid-derived suppressor cells (MDSCs) (12–14). Orchestrated through intricate intercellular crosstalk, these tumor-intrinsic molecular derangements and extrinsic suppressive cellular niches act in concert to construct formidable extracellular matrix (ECM) and chemokine barriers. This synergy effectively precludes the infiltration of effector CD8+ T cells, mediating profound spatial immune exclusion and functional suppression, and ultimately driving the establishment of the immunosuppressive landscape in prostate cancer.

Single-cell RNA sequencing (scRNA-seq) and spatial transcriptomics (ST) enable characterization of cellular heterogeneity and spatial organization in the prostate cancer microenvironment (15–17). Whereas conventional bulk RNA-seq inherently obscures intercellular heterogeneity, single-cell profiling has illuminated a highly diverse yet functionally constrained immune landscape within prostate cancer. A pervasive hallmark woven throughout this complex ecosystem is profound microenvironmental immunosuppression, manifesting as the functional exhaustion or spatial exclusion of effector T cells, concomitantly orchestrated with the coordinated clustering of diverse suppressive stromal elements and immune subpopulations (12, 18). Crucially, spatial omics technologies further unravel the precise geographical proximity and communication logic of these cellular constituents *in situ*, delineating the spatial topography actively sculpted by tumor cells and their surrounding suppressive stroma (19, 20). Systematically deciphering this highly heterogeneous, spatially organized immunosuppressive network not only establishes a mechanistic foundation for elucidating immune tolerance in prostate cancer, but also serves as a critical prerequisite for engineering novel combinatorial strategies aimed at reversing the immune-excluded phenotype and circumventing clinical immunotherapy resistance. Notably, despite their advantages, single-cell RNA sequencing and spatial transcriptomics have technical limitations. Tissue dissociation removes native spatial relationships, can induce stress-related transcriptional changes, and may bias the recovery of specific cell populations (21). Spatial transcriptomic platforms differ in sensitivity and resolution; depending on the method, mixed-cell capture locations and molecular diffusion can complicate the identification of cell states and tissue boundaries (22). Costs associated with sample processing, data generation, and computation can restrict cohort size and longitudinal sampling. Furthermore, differences in tissue handling, platform chemistry, quality control, normalization, integration, and cell annotation complicate comparisons across laboratories (22, 23). Harmonized protocols, transparent reporting, and validation in independent cohorts are therefore needed to improve reproducibility.

### 1.1 Literature search and selection

To ensure the rigor and transparency of this narrative review, a comprehensive literature search was conducted in PubMed, Web of Science, and Scopus for articles published up to October 2026. Only English-language literature was considered. We employed two parallel search strategies. The first focused on microenvironmental profiling, utilizing terms such as “prostate cancer” OR “prostatic neoplasms” combined with “single-cell”, “scRNA-seq”, “spatial transcriptomics”, or “spatial profiling”. The second aimed to capture therapeutic advances, combining prostate cancer terms with “immunotherapy”, “immune checkpoint”, “CAR-T”, “bispecific”, and “clinical trial”.

During study selection, priority was given to peer-reviewed original research and full-text articles involving human prostate cancer cohorts. Studies utilizing murine or canine models were selectively included to elucidate specific molecular mechanisms or preclinical rationales, and the corresponding species are explicitly identified in the text. Peer-reviewed original studies were prioritized; selected conference abstracts and preprints were also considered when they provided relevant emerging mechanistic or clinical evidence. As a narrative review, this manuscript does not claim exhaustive coverage of all existing literature; rather, it selectively synthesizes the most relevant and robust evidence to elucidate the multidimensional immunosuppressive landscape of prostate cancer.

## 2 A panoramic view of the immune microenvironment in prostate cancer

Rather than functioning as a static anatomical entity, the tumor immune microenvironment (TIME) represents a highly dynamic ecosystem that continuously evolves in tandem with tumorigenesis, progression, metastatic dissemination, and therapeutic intervention. Although the immune landscape of prostate cancer exhibits pronounced disease-stage specificity, profound immunosuppression remains the overarching hallmark dictating its entire clinical continuum. Consequently, a comprehensive elucidation of these spatiotemporal dynamics and stage-specific evolutionary trajectories serves as the essential cornerstone for engineering precision immunotherapeutic interventions. To conceptualize this, **Figure 1** delineates the spatiotemporal evolution of the TIME across distinct prostate cancer lineages, highlighting the mechanistic transitions from a relatively immune-infiltrated state in localized disease to profoundly immunosuppressive or immune-desert phenotypes in advanced stages (mCRPC, NEPC, and DNPC).

**Figure 1:**
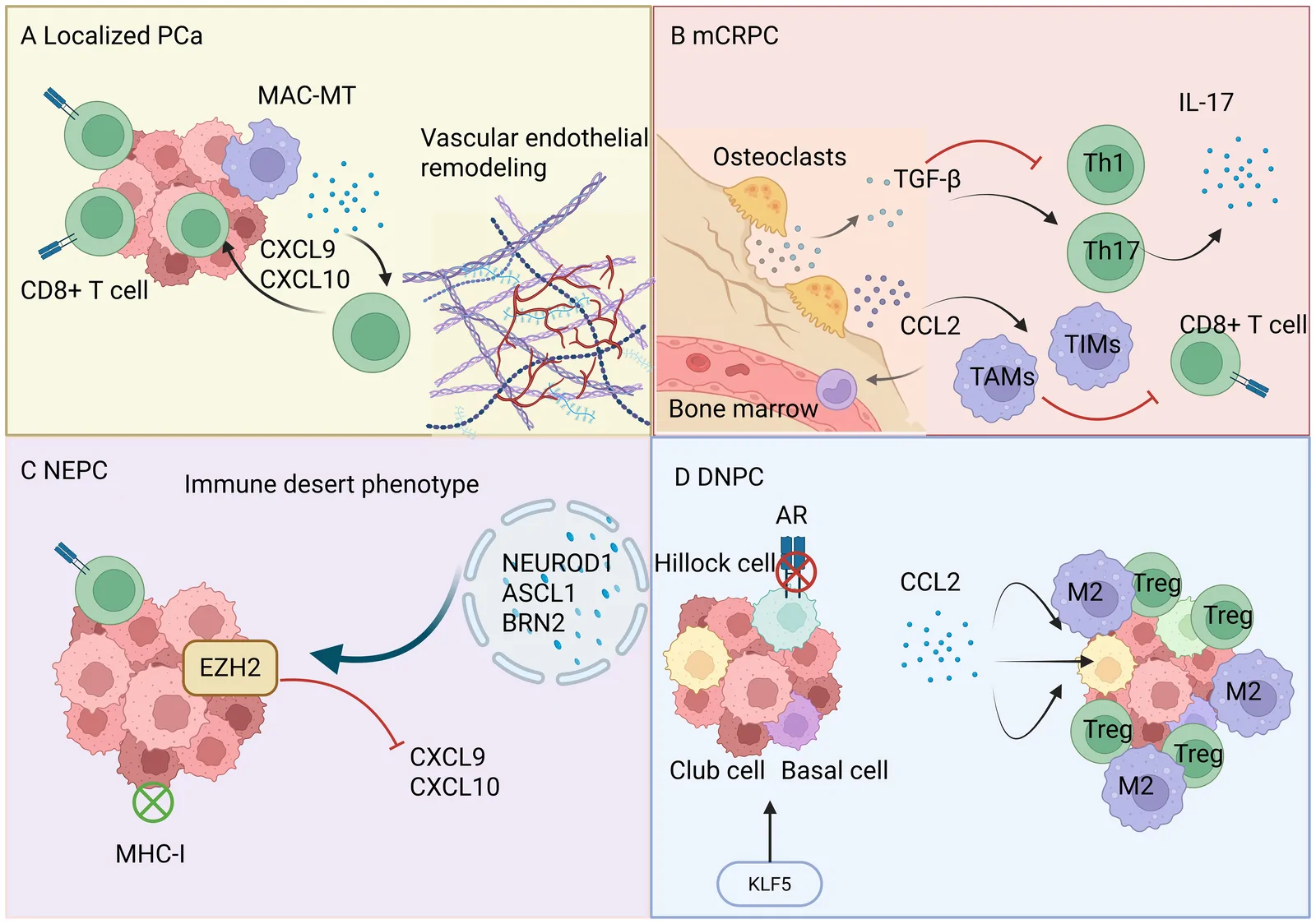
Spatiotemporal evolution of the tumor immune microenvironment across distinct prostate cancer lineages. **(A)** Localized PCa: In the localized prostate cancer stage, specific macrophage subpopulations (e.g., MAC-MT) orchestrate the secretion of chemokines such as CXCL9 and CXCL10, which actively facilitates the recruitment and infiltration of CD8+ T cells. Concurrently, localized vascular endothelial remodeling contributes to a relatively immune-infiltrated microenvironment. **(B)** mCRPC: Upon progression to metastatic castration-resistant prostate cancer, particularly within the bone marrow niche, the microenvironment is profoundly remodeled. Osteoclast-derived TGF-β dampens Th1 cell polarization while promoting Th17 differentiation and subsequent IL-17 secretion. Concomitantly, osteoclast-secreted CCL2 recruits tumor-associated macrophages (TAMs) and tumor-infiltrating myeloid cells (TIMs), which synergistically abrogate the cytolytic activity of CD8+ T cells. **(C)** NEPC: Neuroendocrine prostate cancer typically manifests a distinct “immune desert” phenotype. Neuroendocrine lineage transcription factors (NEUROD1, ASCL1, BRN2) drive the activation of the epigenetic regulator EZH2. This cascade directly represses the expression of MHC-I complexes and T-cell recruiting chemokines (CXCL9/CXCL10), effectively precluding the infiltration of effector T cells. **(D)** DNPC: Double-negative prostate cancer is characterized by the absence of androgen receptor (AR) signaling and the emergence of basal, club, and hillock cell-like phenotypes (modulated by KLF5). These tumor cells robustly secrete CCL2, orchestrating the massive recruitment and spatial accumulation of M2-polarized macrophages and regulatory T cells (Tregs), thereby architecting a highly immunosuppressive localized barrier. PCa, prostate cancer; mCRPC, metastatic castration-resistant prostate cancer; NEPC, neuroendocrine prostate cancer; DNPC, double-negative prostate cancer; MAC-MT, metallothionein-positive macrophages; TAMs, tumor-associated macrophages; TIMs, tumor-infiltrating myeloid cells; AR, androgen receptor; Tregs, regulatory T cells.

### 2.1 Localized prostate cancer immune landscape

Even at the localized stage, prior to macroscopic metastatic dissemination, the prostate tumor microenvironment (TME) already exhibits profound and intricately orchestrated immunosuppressive hallmarks. An integrated single-cell sequencing and spatial transcriptomic analysis of localized prostate cancer following radical prostatectomy revealed that, compared to healthy prostatic tissue, while a modicum of immune cell infiltration is detectable within the primary tumor, the functional integrity of these cells is universally compromised or exhausted (24). Within the lymphoid compartment, infiltrating CD8+ cytotoxic T lymphocytes are not only numerically sparse but also transcriptomically hardwired for early exhaustion, overexpressing a repertoire of inhibitory checkpoints (e.g., PD-1/PDCD1, TIM-3/HAVCR2, and LAG3), thereby severely blunting their antigen-specific cytotoxicity. Concurrently, this landscape is characterized by a significant expansion of immunosuppressive regulatory T cells (Tregs), alongside an enrichment of suppressive myeloid subsets, particularly monocytes and SPP1-expressing tumor-associated macrophages (SPP1+ TAMs). Furthermore, within the treatment-naïve localized TME, the stromal endothelial compartment undergoes profound pro-angiogenic transcriptomic rewiring. Intratumoral endothelial cells significantly upregulate biological pathways governing angiogenesis, vasculature development, and cellular migration, marked by the specific overexpression of endothelial activation and sprouting markers such as ESM1 (endothelial cell-specific molecule 1) and PLVAP (24, 25). This aberrantly remodeled vascular network is intimately intertwined with the evolution of malignant epithelial cells, establishing a critical stromal scaffolding for the construction and maintenance of a localized immunosuppressive niche.

Notably, microenvironmental deconvolution at single-cell resolution unveiled a distinct macrophage subpopulation (MAC-MT) uniquely resident in both healthy human prostates and localized prostate cancer tissues. These macrophages not only highly express an array of metallothioneins but also overexpress zinc transporters (e.g., SLC39A8 and SLC30A1) responsible for orchestrating zinc homeostasis. Given that the prostate constitutes one of the most zinc-enriched organs in the human body, MAC-MTs maintain tissue homeostasis by fine-tuning local zinc ion concentrations under physiological conditions. Crucially, during early primary tumorigenesis, in stark contrast to other myeloid subsets that pervasively adopt immunosuppressive phenotypes, intratumoral MAC-MTs unleash unique anti-tumor potential. They significantly upregulate fatty acid-responsive and pro-inflammatory gene expression programs, actively recruiting T cells and natural killer (NK) cells for local immunosurveillance via the robust secretion of key chemokines such as CXCL9 and CXCL10. Clinical transcriptomic data further corroborate that elevated expression of MAC-MT signature genes within tumor tissues is intimately associated with significantly prolonged disease-free survival in prostate cancer patients (26).

### 2.2 Immune landscape of metastatic castration-resistant prostate cancer

As the disease progresses to the mCRPC stage under the relentless therapeutic pressure of systemic androgen deprivation therapy, the patient’s immune microenvironment undergoes profound remodeling, manifesting a more extreme and intricately suppressive phenotype. Driven by prolonged endocrine therapeutic pressure and the clonal evolution of tumor cells, the tumor microenvironment (TME) in the mCRPC setting exhibits aberrantly hyperactive pro-tumorigenic inflammatory hallmarks alongside a profound enrichment of immunosuppressive microenvironmental factors. Multiple single-cell transcriptomic analyses have revealed that within mCRPC lesions, the anti-tumor Th1-mediated immune response is severely blunted, whereas Th17 cells undergo remarkable enrichment (17, 27, 28). By secreting pro-tumorigenic factors such as interleukin-17 (IL-17), these Th17 cells profoundly potentiate tumor progression and therapeutic resistance (29–31). Crucially, the mCRPC microenvironment—particularly within bone metastases—accumulates elevated levels of transforming growth factor-β (TGF-β) and specific chemokines, which act in concert to drive a profound immunosuppressive network. On one hand, osteoclast-mediated bone matrix resorption unleashes abundant TGF-β, a molecule that potently suppresses the development of anti-tumor Th1 cells and dictates a functional skewing of T helper cells toward the pro-tumorigenic Th17 lineage (27, 32). On the other hand, the tumor-derived chemokine CCL2 robustly recruits peripheral monocyte-macrophages into the bone microenvironment, an infiltration cascade highly contingent upon the degradation and remodeling of the bone collagen matrix by macrophage-derived cathepsin K (CTSK) (33). Upon infiltration, these monocytes are locally driven to differentiate into tumor inflammatory monocytes (TIMs), M2-polarized tumor-associated macrophages (TAMs), and osteoclasts. Within an intricately communicating network, these cells establish a positive feedback loop that further amplifies the secretion of inflammatory mediators such as CCL2 and COX-2, thereby potentiating local tumor growth while concomitantly exacerbating osteolysis; subsequently, these aberrantly enriched and phenotypically skewed TIMs and TAMs locally secrete copious amounts of the chemokine CCL20, which directly exacerbates the terminal exhaustion of tumor-infiltrating CD8+ T cells via the CCL20-CCR6 signaling axis (28, 34). Furthermore, the TME robustly recruits polymorphonuclear myeloid-derived suppressor cells (PMN-MDSCs) into the tumor core. The massive infiltration of these cells is highly correlated with the spatial exclusion and functional suppression of CD8+ T cells, constituting a fundamental cornerstone of immunotherapy resistance in mCRPC (35, 36).

### 2.3 Neuroendocrine and double-negative prostate cancer immune landscape

Under the chronic selective pressure of potent androgen receptor pathway inhibitors (ARPIs, such as enzalutamide and abiraterone), a fraction of prostate cancer cells entirely circumvent their androgen receptor (AR) dependency via lineage plasticity. Through transdifferentiation or dedifferentiation, they evolve into highly aggressive variant subtypes with dismal prognoses, namely neuroendocrine prostate cancer (NEPC) and double-negative prostate cancer (DNPC).

Along the evolutionary trajectory of ARPI resistance, NEPC exhibits profound immune evasion characteristics, clinically and pathologically presenting as a canonical “immune desert” phenotype (37). Within the microenvironmental evolution of NEPC, genomic alterations driven by lineage plasticity and epigenetic rewiring act in concert to establish its extreme immune-evasive hallmarks. The transdifferentiation process is mediated by neuronal differentiation factors (e.g., NEUROD1, ASCL1, and BRN2) and is fundamentally driven by the upstream concurrent loss of function of TP53 and RB1, alongside MYCN gene amplification (38). Bhinder et al. further demonstrated that transcriptional pathways associated with the G2/M cell cycle checkpoint and E2F targets are significantly upregulated in NEPC; the aberrant activation of these pathways not only endows the tumor with exceptionally high proliferative activity but also profoundly potentiates its invasive capabilities (37). Concurrently, the secretome remodeling and metabolic adaptation (such as 5-HT release and aberrant surface glycosylation) that accompany this lineage conversion rewire the microenvironmental networks of distant organs, thereby conferring upon this subtype a strong propensity for visceral colonization and metastasis (e.g., liver metastasis) (39, 40). Epigenetically, the polycomb repressive complex 2 (PRC2), featuring EZH2 as its catalytic subunit, exhibits a state of pronounced transcriptional activation in NEPC. By mediating H3K27me3 modifications, it triggers genome-wide chromatin restructuring, driving the transdifferentiation of the tumor toward the neuroendocrine lineage and silencing tumor-suppressive programs (41). This epigenetic reprogramming dampens tumor immunogenicity at its source by repressing MHC-I molecules and antigen processing and presentation machinery (APM)-associated genes. Simultaneously, the elevated EZH2 activity synergistically suppresses the transcription of key effector T-cell recruitment factors (such as CXCL9 and CXCL10), effectively paralyzing the chemotactic network of the tumor microenvironment. Furthermore, single-cell multi-omics analyses have revealed that EZH2 remodels microenvironmental cellular communication; by reinforcing the suppressive crosstalk between the tumor and regulatory T cells (Tregs), it spatially reconfigures the immune-tolerant landscape of the TME (42). This paralysis of the chemotactic network and defect in antigen presentation, orchestrated by tumor-intrinsic epigenetic regulation, ultimately cause metastatic lesions to manifest as canonical “immune deserts”. Although a fraction of NEPC cells may undergo compensatory upregulation of PD-L1 driven by endogenous oncogenic pathways, the profound spatial paucity of effector lymphocyte infiltration renders this subtype broadly and primarily resistant to systemic anti-tumor regimens, including chemotherapy, targeted therapies, and conventional PD-1/PD-L1 inhibitors (37). The canonical pathological hallmark of DNPC is defined by the concurrent absence of androgen receptor (AR) expression and traditional neuroendocrine markers, such as synaptophysin (SYP) and chromogranin A (CHGA) (43, 44). Epigenomic profiling integrating bulk RNA sequencing (RNA-seq) and assay for transposase-accessible chromatin using sequencing (ATAC-seq) has further elucidated the spatial and molecular heterogeneity intrinsic to DNPC, establishing two novel therapy-resistant subpopulations that are starkly distinct in their transcriptomic profiles and chromatin accessibility landscapes: the CRPC-WNT subtype and the stem cell-like (CRPC-SCL) subtype. Notably, the CRPC-WNT subtype is predominantly driven by the TCF/LEF transcription factor family, frequently accompanied by somatic mutations or epigenetic activation of genes implicated in the WNT signaling cascade. In stark contrast, the CRPC-SCL subtype, which comprises approximately 20% of the overall CRPC population, is synergistically orchestrated by the YAP/TAZ/TEAD complex and AP-1 family members (specifically FOSL1). Within this subtype, FOSL1 acts as a pivotal transcription factor that rewires chromatin accessibility, engaging in a positive feedback loop with the YAP/TAZ pathway to coordinately drive highly enriched prostate basal stem cell transcriptomic signatures; this ultimately culminates in a significantly curtailed time to progression in patients receiving novel androgen receptor pathway inhibitor (ARPI) therapies (45).

Interestingly, comparative transcriptomic analyses at single-cell resolution have corroborated that individual DNPC tumor cells co-express a mixed epithelial identity encompassing basal, club, and hillock cellular features, with the transcription factor KLF5 playing a pivotal role in perpetuating this mixed differentiation state (46). Regarding its immunological microenvironment, DNPC exhibits an intricate multicellular crosstalk network profoundly sculpted by an aberrant secretome (47). First, the master epigenetic regulator polycomb repressive complex 1 (PRC1) undergoes pathological activation in DNPC; by potently inducing tumor cells to secrete the chemokine CCL2, it establishes a critical paracrine immunosuppressive loop. Elevated levels of CCL2 not only drive the stemness and self-renewal of tumor cells but also directionally recruit an abundance of CCR2-expressing M2-polarized tumor-associated macrophages (TAMs) and Tregs directly into the tumor core (47). Moreover, this microenvironmental suppressive network exhibits extraordinary spatial and multicellular interactive complexity. Spatial transcriptomics reveals that epithelial subpopulations harboring club cell features within DNPC release multiple chemokines via the senescence-associated secretory phenotype (SASP), robustly recruiting polymorphonuclear myeloid-derived suppressor cells (PMN-MDSCs) and thereby exacerbating localized, myeloid-driven immune exclusion (19). At the level of stromal crosstalk, tumor-derived CCL2 stimulates microenvironmental fibroblasts to secrete FGF8b, which subsequently undergoes paracrine signaling to pathologically hyperactivate the intrinsic KRAS/MAPK signaling axis within tumor cells, further sustaining AR-independent malignant clonal expansion (48). Within heterogeneous subpopulations exemplified by CRPC-WNT, tumor cells also significantly blunt the cytolytic activity of natural killer (NK) cells through the secretion of soluble factors such as Dickkopf-1 (DKK-1) (49). Ultimately, these aberrant cytokine secretions severely constrain the infiltration of effector cells such as CD8+ T cells, firmly establishing the pathological foundation for primary immune resistance in DNPC.

## 3 Analysis of immunosuppressive cell components

The canonical “immune-cold” tumor phenotype of prostate cancer can by no means be fully elucidated by the singular epiphenomenon of constrained T-cell infiltration. Fundamentally, its essence is rooted in a highly complementary, immunosuppressive ecological network intricately woven by diverse immune and stromal populations within the tumor microenvironment. The rapid evolution and integration of single-cell sequencing and spatial transcriptomics have empowered researchers to deconstruct the subclonal heterogeneity, spatial distribution topography, and intricate intercellular communication circuitries of these suppressive cellular niches at in greater detail. **Figure 2** delineates the comprehensive cellular landscape of this microenvironment, mapping the synergistic mechanisms by which major stromal and immune populations—specifically CAFs, TAMs, MDSCs, and Tregs—converge to orchestrate multifarious immune evasion pathways and abrogate effector CD8+ T-cell functionality.

**Figure 2:**
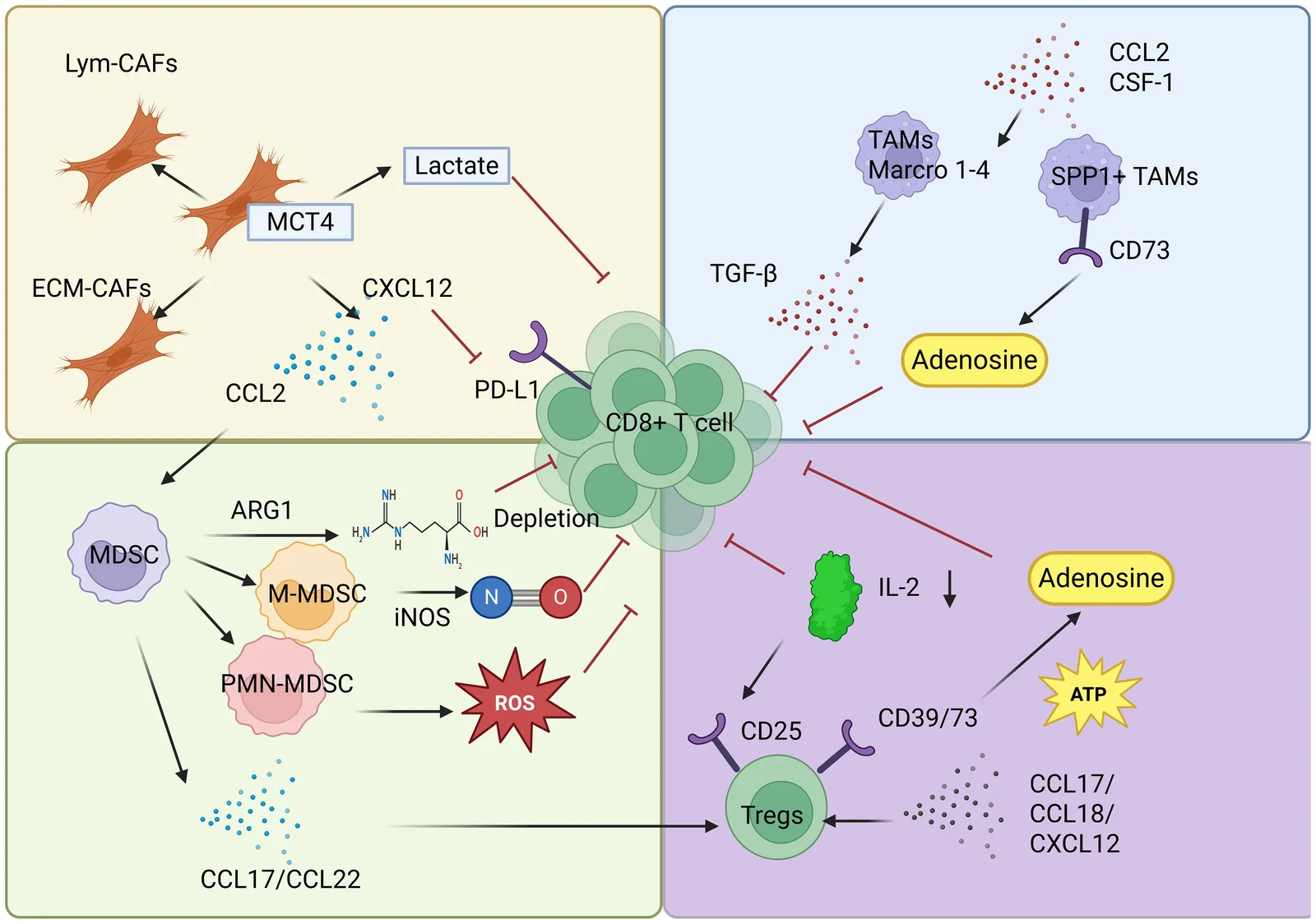
The cellular landscape of the prostate cancer immune microenvironment. The schematic illustrates the synergistic mechanisms by which diverse stromal and immune cell populations orchestrate immune evasion, converging on the suppression of effector CD8+ T cells. Top Left: Cancer-associated fibroblasts (CAFs) exhibit phenotypic plasticity between extracellular matrix-associated CAFs (ECM-CAFs) and lymphocyte-associated CAFs (Lym-CAFs). They mediate immunosuppression through lactate accumulation (facilitated by MCT4), the secretion of chemokines (CXCL12, CCL2), and the upregulation of the immune checkpoint molecule PD-L1. Top Right: Tumor-associated macrophages (TAMs), recruited by CCL2 and CSF-1, encompass diverse subpopulations (Macro 1-4). The SPP1+ TAM subset notably drives the accumulation of immunosuppressive adenosine via CD73. Additionally, TAM-derived TGF-β directly abrogates T-cell effector functions. Bottom Left: Myeloid-derived suppressor cells (MDSCs) stratify into monocytic (M-MDSC) and polymorphonuclear (PMN-MDSC) subtypes. They blunt CD8+ T cell activity via enzymatic metabolic depletion (ARG1), nitric oxide (NO) production, and reactive oxygen species (ROS) generation. MDSCs further amplify immunosuppression by secreting CCL17 and CCL22 to recruit Tregs. Bottom Right: Regulatory T cells (Tregs), recruited by local chemokine gradients (CCL17/CCL18/CXCL12), competitively sequester interleukin-2 (IL-2) via the high-affinity CD25 receptor, precipitating the metabolic deprivation of CD8+ T cells. Furthermore, Tregs hydrolyze pro-inflammatory ATP into suppressive adenosine through the CD39/CD73 metabolic axis. CAFs, cancer-associated fibroblasts; Lym-CAFs, lymphocyte-associated CAFs; ECM-CAFs, extracellular matrix-associated CAFs; MCT4, monocarboxylate transporter 4; TAMs, tumor-associated macrophages; SPP1, secreted phosphoprotein 1; MDSC, myeloid-derived suppressor cell; M-MDSC, monocytic MDSC; PMN-MDSC, polymorphonuclear MDSC; ARG1, arginase 1; NO, nitric oxide; ROS, reactive oxygen species; Tregs, regulatory T cells; ATP, adenosine triphosphate.

### 3.1 TAMs

The prostate cancer microenvironment is hallmarked by profound immunosuppression, within which bone marrow-derived tumor-associated macrophages (TAMs) constitute the most predominant immune cell subpopulation in the stromal compartment. Tumor cells and their pro-fibrotic stroma specifically upregulate the secretion of CCL2 and macrophage colony-stimulating factor (CSF-1) to orchestrate the recruitment of TAMs (50, 51). By secreting cytokines such as vascular endothelial growth factor (VEGF) and TGF-β, these TAMs actively participate in sculpting the immunosuppressive microenvironment of prostate cancer (8, 52).

Traditional paradigms have largely relied on a simplified *in vitro* M1 (anti-tumor)/M2. (pro-tumor) binary polarization model to conceptualize TAMs. However, propelled by the intersection of single-cell RNA sequencing (scRNA-seq) and spatial transcriptomics, researchers have now recognized that prostate cancer TAMs do not adhere to this simplistic M1/M2 dichotomy, but rather exist in a highly continuous transcriptomic state. In 2025, Lyu et al. delineated a distinct TAM subpopulation characteristically exhibiting high SPP1 expression (SPP1hi-TAMs). Concomitant with the progression of prostate cancer toward mCRPC, both the abundance of this subpopulation and the immunosuppressive molecular programs it harbors exhibit a profoundly escalating trajectory.

It is particularly noteworthy that, across both human and murine prostate cancer models, these SPP1+ TAMs specifically overexpress the ectonucleotidase CD73 (encoded by the NT5E gene) relative to other myeloid subsets. Notably, during the mCRPC stage, both the transcriptional levels of NT5E and the *in vivo* CD73 protein levels within SPP1hi-TAMs undergo pronounced upregulation. The hypoxic tumor microenvironment drives these CD73-high SPP1+ TAMs to robustly catalyze and release extracellular adenosine, thereby constructing a potent immunosuppressive niche via the adenosine signaling pathway.

Furthermore, based on high-throughput sequencing analyses by May et al. in 2025, TAMs. in prostate cancer can be principally categorized into four highly specific functional and phenotypic subpopulations (Macro-1 through Macro-4) (17). Segregating specialized roles based on their unique transcriptomic signatures, spatial niche localizations, and functional capacities to drive malignant progression and therapeutic resistance, these subpopulations act in concert to weave the intricate immune evasion and metastatic network of prostate cancer.

#### 3.1.1 Tissue residency and antigen-presenting subgroup (Macro-1)

Within the prostate cancer immune microenvironment, tissue-resident macrophages (TRMs) of embryonic origin (e.g., yolk sac or fetal liver) exert a pivotal regulatory role in mediating tumor immune evasion and microenvironmental remodeling. This subpopulation is primarily characterized by the high expression of folate receptor beta (FOLR2), selenoprotein P (SEPP1), and MHC class II antigen presentation-associated molecules (such as CD74 and HLA-DRA) (17). By highly expressing MHC class II-associated molecules and robustly activating antigen processing and presentation pathways, the Macro-1 subpopulation epitomizes the anti-tumor immunosurveillance function of FOLR2+ TRMs during early tumorigenesis. This subpopulation can efficiently process tumor antigens and cross-present them to T cells, thereby acting as a central hub in priming specific adaptive anti-tumor immune responses. Although the precise spatial distribution of these FOLR2+ tissue-resident macrophages within the prostate cancer microenvironment awaits further elucidation, multiple studies across other solid tumors indicate that they possess highly conserved spatial localization hallmarks. Indeed, across diverse malignancies including pancreatic cancer (53), hepatocellular carcinoma (54), and breast, colorectal, and lung cancers (55, 56), FOLR2+ macrophages are consistently detected in close apposition to the vasculature within the tumor stroma, thus being specifically defined as perivascular tissue-resident macrophages. Notably, these studies further reveal that the secretion of inhibitory cytokines such as IL-10, TGF-β, and IL-18 by these cells not only directly abrogates effector T-cell responses but also promotes the differentiation of terminally differentiated effector Tregs, constructing a profound immunosuppressive network (57). Furthermore, Macro-1 cells residing within the perivascular niche release copious amounts of VEGF-A and PDGF, driving aberrant angiogenesis and establishing a permissive condition for metastatic dissemination (58). This might elucidate the profoundly suppressive nature of the prostate cancer immune microenvironment.

#### 3.1.2 Central subgroup of chemotactic network (Macro-2)

Similar to the Macro-1 subpopulation, Macro-2 shares the tissue-resident hallmarks of high FOLR2 and SEPP1 expression. However, its core molecular phenotype is distinguished by the pronounced upregulation of the chemokine CCL2 alongside early activation response transcription factors such as FOS and EGR1, extending beyond the baseline features of FOLR2+ macrophages. FOS and EGR1 are canonical immediate-early genes and pivotal transcription factors, the expression of which is typically rapidly upregulated upon exposure to microenvironmental physicochemical stimuli, including hypoxia, oxidative stress, or localized inflammation. This suggests that Macro-2 cells reside in a highly active state of early activation or transcriptional stress, endowing them with the capacity to acutely sense dynamic fluctuations within the tissue microenvironment. Studies have demonstrated that EGR1 within macrophages can recruit the NuRD corepressor complex to target and repress the activity of inflammatory enhancers, thereby actively blunting the pro-inflammatory responses of these cells (59). Moreover, within the tumor niche, CCL2 not only persistently chemoattracts peripheral blood monocytes to the local site, but these recruited cells also exhibit a strong propensity to differentiate into immunosuppressive M2-polarized macrophages, further potentiating tumor invasion and metastasis (60). Collectively, through the synergistic interplay of EGR1-mediated intrinsic anti-inflammatory transcriptional repression and CCL2-driven extrinsic suppressive network remodeling, the Macro-2 subpopulation acts in concert to drive the maintenance and exacerbation of the localized immunosuppressive microenvironment.

#### 3.1.3 Pro-inflammatory macrophage subset (Macro-3)

Transcriptomically, the Macro-3 subpopulation exhibits a profound resemblance to canonical M1-polarized macrophages. It is distinguished not only by the robust overexpression of core pro-inflammatory genes, including NLRP3, TNFAIP3, and IL1B, but also by possessing the highest M1 signature and inflammasome activation scores. Nevertheless, evaluations of macrophage spatial topography reveal that the highly inflammatory Macro-3 subpopulation (IL1B+, NLRP3+) is predominantly sequestered within tumor-adjacent normal tissue regions. This compartmentalization stands in stark contrast to metastasis-associated macrophages (Macro-4: SPP1+, TREM2+, APOE+), which manifest pronounced spatial clustering directly within the tumor interior (17). This unique spatial disparity dictates that despite harboring robust pro-inflammatory hallmarks and potent anti-tumor reactivity, Macro-3 cells fail to effectively infiltrate the core tumor microenvironment. Such physical spatial exclusion likely abrogates their capacity for direct immunosurveillance and cytolytic eradication of the malignant nest.

#### 3.1.4 Ultimate transfer-driven subpopulation (Macro-4)

Relative to other subsets, the Macro-4 subpopulation has been subjected to more in- depth investigation. Macro-4 represents an M2-polarized macrophage subset endowed with profound pro-tumorigenic and immunosuppressive properties. Its core transcriptomic hallmark is distinguished by the pronounced overexpression of TREM2, SPP1, APOE, and GPNMB, concomitantly exhibiting a compromised antigen presentation capacity. Spatially, in stark contrast to inflammatory macrophages that are sparsely dispersed within normal tissue regions, Macro-4 exhibits pronounced intratumoral spatial clustering, specifically and deeply infiltrating the core regions of primary tumors as well as distant metastatic niches such as bone metastases.

Mechanistically, transcriptomic profiling and Gene Set Enrichment Analysis (GSEA) intimate that this subpopulation harbors the transcriptional potential to architect a pro-tumorigenic microenvironment. The upregulation of genes such as MMP9, MMP14, and MMP19 implies a potent capacity for extracellular matrix (ECM) remodeling; meanwhile, the elevated expression of angiogenesis-associated genes (e.g., VEGFB) and epithelial-mesenchymal transition (EMT)-related genes (e.g., VIM and FN1) reflects its putative role in orchestrating tumor invasion and tissue remodeling. This transcriptome-based deduction has received preliminary biological corroboration in murine intervention models: although the targeted depletion of the SPP1-expressing Macro-4 population did not manifest a direct tumor-suppressive effect as a monotherapy, it effectively potentiated the local infiltration of CD8+ T cells, thereby remodeling the immune microenvironment and significantly sensitizing tumors to PD-1 immune checkpoint blockade (17). Furthermore, the high levels of SPP1 expressed by Macro-4 further induce the M2 polarization of macrophages within the localized microenvironment. Concurrently, the robust expression of CCL18—a chemokine previously proven to drive metastasis in breast, colon, and squamous cell carcinomas—further accentuates the potential role of Macro-4 in potentiating tumor dissemination (61, 62). Notably, research has also revealed that Macro-4 is significantly more enriched in metastatic specimens compared to localized tumor samples (17). These findings collectively suggest that across multiple dimensions—encompassing the impairment of antigen presentation, promotion of angiogenesis, and modulation of the ECM—Macro-4 acts in concert to drive the invasion, metastasis, and immune evasion of malignancies such as prostate cancer.

### 3.2 Tregs

Regulatory T cell (Treg) infiltration into prostate cancer tissues is an active process driven by specific chemokine gradients secreted by tumor, stromal, and other immune cells. Furthermore, these chemokine-receptor interactions regulate local Treg accumulation and promote a therapy-resistant microenvironment (63). Among the multiple recruitment pathways, the CCL17/CCL22-CCR4 axis exerts a predominant role. In spontaneous canine prostate cancer, Maeda et al. reported increased expression of C-C motif chemokine ligand 17 (CCL17). Dogs harboring the BRAF V595E mutation had higher urinary CCL17 concentrations and greater tumor infiltration by regulatory T cells than dogs with wild-type BRAF (64). Beyond this dominant chemotactic pathway, tumor cells and stromal cells within the prostate cancer microenvironment can also secrete the chemokine CCL28, which synergistically mediates the infiltration of Tregs into tumor tissues by specifically engaging the CCR10 receptor on their surface. This non-canonical chemotactic mechanism exerts a pronounced compensatory role during Treg recruitment (65). In addition to tumor and stromal cells, tumor-associated macrophages (TAMs) play a pivotal role in recruiting Tregs. Single-cell and spatial omics have unveiled that by secreting CXCL12, macrophages leverage the CXCL12-CXCR4 receptor-ligand interaction to co-recruit CXCR4-expressing Tregs and CD8+ effector T cells into a shared spatial niche. This architectural proximity provides the requisite spatial prerequisites for Tregs to induce CD8+ T-cell exhaustion through the competitive sequestration of local IL-2 (65). Consequent to the application of multiplex immunofluorescence (cycIF) and spatial transcriptomics, the precise spatial topography of Tregs within the microenvironment has been progressively elucidated. Within the tissue architecture, Tregs manifest a highly structured “cellular neighborhood network”, wherein their suppressive efficacy is profoundly contingent upon their physical proximity to target cells (66). In high-grade prostate cancer (e.g., Grade 3 and 4), subsets of mature tertiary lymphoid structures (TLS) exhibit an exceptionally high density of Tregs in intimate spatial apposition with exhausted T cells (67). This extreme physical proximity empowers Tregs to potently blunt the cytolytic functions of effector T cells via contact-dependent mechanisms. Concurrently, the heterogeneity of stromal cells exerts a pronounced impact on the spatial distribution of Tregs. Spatial colocalization analyses indicate that androgen receptor (AR)-expressing mast cells exhibit a strong propensity for highly enriched spatial interactions with Tregs; in stark contrast, granzyme B-expressing mast cells predominantly colocalize with M2-polarized macrophages (68). Through spatial co-localization with macrophages and mast cells, Tregs establish a distinct immunosuppressive niche.

Beyond the occupation of physical space, the predominant immune-subversive mechanism of Tregs lies in their “predatory metabolic competition” for critical survival factors, coupled with the copious generation of suppressive metabolites (65). Foremost among these is the competitive sequestration of interleukin-2 (IL-2). IL-2 is an indispensable cytokine for sustaining T-cell clonal expansion and cytotoxicity. Tregs constitutively overexpress the high-affinity IL-2 receptor complex (CD25) on their surface, yet intrinsically lack the transcriptional capacity to produce IL-2 (69, 70). Facilitated by macrophage-mediated co-recruitment, spatially adjacent Tregs are capable of competitively depleting local IL-2, thereby robustly hyperactivating the IL-2/STAT5 signaling cascade internally. This metabolic deprivation drives the functional exhaustion of neighboring CD8+ effector T cells, subsequently inducing their forward transcription and pronounced surface upregulation of PD-1 (PDCD1) and PD-L1 (CD274)—an adaptive immunosuppressive phenotype that is notably absent in canonical tissue-resident T cells (65).

Beyond the competitive sequestration of cytokines, Tregs also exert multidimensional immunosuppressive functions within the hypoxic tumor microenvironment via the CD39/CD73 metabolic regulatory axis. Specifically, the ectoenzymes CD39 and CD73, which are highly expressed by Tregs, act in concert to hydrolyze pro-inflammatory extracellular ATP into adenosine (71). The localized accumulation of adenosine directly abrogates the activation of effector T cells and NK cells by engaging specific receptors on their surface (72). In summary, Tregs within the prostate cancer microenvironment are actively recruited via multiplexed chemotactic networks to establish highly specific spatial niches; subsequently, through mechanisms encompassing spatial proximity-dependent contact, competitive IL-2 sequestration, and adenosine metabolic modulation, they synergistically abrogate the function of localized effector T cells.

### 3.3 CAFs

CAFs within the prostate cancer microenvironment do not originate from a singular cellular lineage; rather, their diverse ontogeny underpins their profound functional heterogeneity. Senescent normal stromal fibroblasts constitute a crucial precursor reservoir for CAFs (73). Upon stimulation by tumor-derived growth factors and inflammatory cytokines, these senescent fibroblasts not only evade apoptosis but also secrete paracrine factors to drive the aberrant proliferation of prostate epithelial cells, concurrently transdifferentiating into CAFs (74). Furthermore, in estrogen-overexpressing prostate cancer models, innate immune cells such as mast cells and macrophages can unleash tryptase and IL-13, which independently stimulate the proliferation and transdifferentiation of CAFs by circumventing the canonical TGFβ2-STAT6 signaling axis (75). In orchestrating the remodeling of the immunosuppressive prostate cancer microenvironment, CAFs exhibit profound phenotypic heterogeneity and multidimensional regulatory capacities. Based on single-cell transcriptomic (scRNA-seq) profiling, CAFs within prostate cancer tissues can be principally stratified into two functional subpopulations: extracellular matrix-associated CAFs (ECM-CAFs) and lymphocyte-associated CAFs (Lym-CAFs). The ECM-CAF phenotype is predominantly induced by TGF-β signaling and governed by YAP1 transcriptional activity; this subset characteristically overexpresses markers such as CD248, COL1A1, COL1A2, CNN1, and CCN2, driving prostate cancer progression primarily by potentiating collagen deposition and extracellular matrix remodeling. Conversely, the Lym-CAF phenotype is induced by the synergistic stimulation of TNF-α and IFN-γ, orchestrated by the core transcription factor NF-κB p65. This subpopulation uniquely overexpresses TNFAIP6, IL33, and an array of chemokines and cytokines (e.g., CXCL8, CXCL9, CXCL10, and CXCL11), exerting an anti-tumor immunomodulatory function that facilitates the infiltration and activation of CD8+ T cells (76). Despite their stark functional dichotomy, these two CAF subsets share profound phenotypic plasticity and spatial regulatory commonalities: their phenotypic transitions are intricately choreographed by master regulators such as YAP1. The activation of YAP1 sustains the ECM-CAF phenotype and promotes collagen deposition, whereas its knockdown or silencing can drive a transdifferentiation toward the Lym-CAF state, a dynamic that plays a pivotal role in the evolutionary trajectory of the tumor stroma (76).

Beyond phenotypic evolution, metabolic rewiring and multidimensional cellular crosstalk act in concert to constitute the core axis of CAF-mediated immune evasion. Metabolically, a reciprocal, bidirectional metabolic reprogramming exists between prostate cancer cells and CAFs. Upon contact with malignant cells, CAFs undergo a metabolic shift hallmarked by the “Warburg effect”. During this process, CAFs upregulate the glucose transporter GLUT1 to enhance glucose uptake, generating copious amounts of lactate, and rely on the *de novo* expression of monocarboxylate transporter 4 (MCT4) to shuttle lactate into the extracellular space. Concomitantly, adjacent prostate cancer cells pivot toward aerobic metabolism; they downregulate GLUT1 expression and become highly reliant on monocarboxylate transporter 1 (MCT1) to avidly sequester the CAF-derived lactate. This internalized lactate is channeled into the mitochondrial tricarboxylic acid (TCA) cycle of the cancer cells, serving as an energy-rich substrate to fuel their oxidative phosphorylation, anabolism, and cellular proliferation, thereby progressively emancipating them from glucose dependency (77). This metabolic coupling not only competitively deprives T cells of the glucose requisite for their activation and clonal expansion but also acidifies the microenvironment via excessive lactate release. Furthermore, it synergistically upregulates the ectoenzyme CD73 to catalyze the conversion of extracellular ATP into high concentrations of adenosine, which directly engages receptors on effector T cells to abrogate their cytolytic programs (78, 79).

At the level of cellular communication, CAFs mediate immunosuppression within the tumor microenvironment (TME) via multiple paracrine and chemotactic cascades. Firstly, cytokines secreted by CAFs, such as TGF-β, IL-10, and IL-6/8, directly downregulate the release of perforin and IFN-γ by infiltrating T cells, severely blunting their cytotoxic functions (80). Moreover, these paracrine factors induce the upregulation of programmed death-ligand 1 (PD-L1) on both tumor and stromal cells, which subsequently triggers the anergy and exhaustion of effector T cells via the PD-1/PD-L1 signaling axis (80). Secondly, CAFs secrete high levels of the chemokine CXCL12; upon engaging its specific receptor CXCR4, this factor establishes a spatial and chemotactic barrier at the periphery of the tumor stroma, effectively precluding the infiltration of cytolytic CD8+ T cells. Concurrently, this chemotactic axis facilitates the directional recruitment of Tregs and MDSCs into the tumor tissue (81). CAFs expressing fibroblast activation protein (FAP) orchestrate a central chemotactic and recruiting role. Specifically, through a uPAR-dependent FAK-Src-JAK2 signaling cascade, CAFs constitutively hyperactivate the intracellular transcription factor STAT3. This fibroblast-intrinsic STAT3 activation directly drives the robust expression and secretion of the chemokine CCL2. Subsequently, the elevated local concentration of CCL2 engages its specific receptor CCR2, directionally and massively recruiting MDSCs from the peripheral circulation into the TME, thereby laying the foundation for the establishment of an immunosuppressive state (82). Ultimately, this multidimensional cellular crosstalk orchestrated by CAFs perpetuates a stably immunosuppressed localized tumor microenvironment.

### 3.4 MDSCs

The recruitment of MDSCs into the tumor parenchyma and stromal compartments of prostate cancer constitutes a complex physiological process intricately orchestrated by multiple chemokine axes, specific genomic alteration landscapes, and microenvironmental physicochemical stress. A myriad of chemokine receptors and their respective ligands participate in MDSC mobilization, encompassing the CCL2/CCL12-CCR2 axis that mediates the mobilization of monocytic MDSCs (82), as well as the CCL3/CCL4/CCL5-CCR5 axis which is implicated in microenvironmental remodeling (83). Aberrations in intrinsic signaling pathways within specific prostate cancer cells have also been observed to correlate with altered secretory profiles of microenvironmental chemokines. For instance, the pathological hyperactivation of the Hippo-YAP signaling pathway is accompanied by the upregulation of CXCL5 expression in tumor cells, which subsequently mediates MDSC chemotaxis via the CXCL5-CXCR2 axis (84, 85). Furthermore, models of PTEN tumor suppressor inactivation exhibit a profound inflammatory propensity, accompanied by the excessive secretion of factors such as colony-stimulating factor-1 (CSF-1) and IL-1β; these alterations are consistent with the pervasive infiltration of MDSCs within the microenvironment (86). Similarly, the loss of the chromatin remodeling protein CHD1 has been suggested to influence MDSC recruitment through an IL-6-dependent mechanism (87, 88), and intratumoral CHD1 levels exhibit a positive correlation with CD15, a marker representative of polymorphonuclear MDSCs (PMN-MDSCs) (88). Beyond intrinsic tumor genomic hallmarks, extrinsic pressures and clinical interventions also profoundly impact the dynamic homeostasis of MDSCs within the microenvironment. The characteristically hypoxic milieu of tumor tissues is recognized as a microenvironmental factor that sustains the undifferentiated state of MDSCs and facilitates their intralesional accumulation (89). In observations of therapeutic interventions, following the application of targeted radionuclide therapy (TRT), model tumor cells exhibit an adaptive secretion of CXCL1 and CXCL2, concomitant with a significant escalation in MDSC abundance within the microenvironment. Conversely, in sequential treatment models where androgen deprivation therapy (ADT) is administered prior to TRT, microenvironmental signaling shifts promote a relative increase in the infiltration of activated phenotype CD8+ and CD4+ T cells (90). Concurrently, systemic psychoneuroendocrine alterations, such as fluctuating secretion levels of neuropeptide Y (NPY), may also participate in this process (91).

Upon infiltrating and residing within the tumor microenvironment, MDSCs exert multidimensional roles in restricting immune responses through diverse enzymatic metabolic competition and the secretion of inhibitory mediators. MDSCs are primarily classified into heterogeneous subpopulations, including monocytic (M-MDSCs), polymorphonuclear/granulocytic (PMN-MDSCs), and early-stage (e-MDSCs), each exhibiting distinct mechanistic signatures in executing their effector functions (92). The pathological activation and immunosuppressive functions of MDSCs are intimately governed by key transcription factors such as STAT3. In manifesting immunomodulatory activity, MDSCs (particularly the M-MDSC subpopulation) rely predominantly on the activity of inducible enzymes like arginase-1 (ARG1). By metabolically consuming and converting L-arginine within the local microenvironment, these enzymes precipitate a profound depletion of this essential amino acid, thereby effectively abrogating normal T-cell proliferation and the expression of immune functions (92). Clinical observations of patients with localized prostate cancer experiencing severe fatigue following external beam radiation therapy (EBRT) reveal that elevated ARG1 expression in peripheral blood is associated not only with a decline in absolute lymphocyte counts but also exhibits a statistical interaction with the expression of T-cell proliferation markers (e.g., CD8, CD28, CD27, CCR7) (93). Furthermore, in analyses of human prostate cancer progression, a specific granulocytic MDSC subpopulation bearing the Lin (–)CD15(HI)CD33(LO) phenotype was found to be significantly enriched within both the tumor microenvironment and the peripheral circulation. This cellular population, characterized by high levels of activated STAT3, primarily relies on the STAT3-ARG1 signaling axis to blunt the proliferative capacity of autologous CD8+ T cells and their anti-tumor activity mediated by the secretion of interferon-γ (IFNγ) and granzyme-B. Additionally, these MDSCs express Toll-like receptor 9 (TLR9) on their surface, a feature that enables them to serve as an effective conduit for the targeted delivery of STAT3 siRNA, thereby offering a potential interventional strategy to reverse immunosuppression within the tumor microenvironment (94). Comparatively, M-MDSCs more heavily exploit the high expression of inducible nitric oxide synthase (iNOS) and the copious production of nitric oxide (NO) to exert non-antigen-specific immunosuppression. Regarding core biochemical and molecular hallmarks, Bronte et al. have clearly delineated the mechanistic divergences between the two subpopulations: M-MDSCs are characterized by high NO and low levels of reactive oxygen species (ROS) and peroxynitrite (PNT); conversely, PMN-MDSCs predominantly rely on generating high levels of ROS and PNT to exert antigen-specific immunosuppression. Despite this dichotomy in ROS and NO production, both major subpopulations share the capacity to co-express arginase-1 (ARG1), effectively blunting effector T-cell activity through distinct biochemical regulatory pathways (92).

Notably, MDSCs engage in extensive crosstalk and synergy with other cellular populations within the microenvironment, among which Tregs constitute a pivotal collaborative cohort. Clinical observations in prostate cancer patients reveal a phenomenon of co-enrichment of Tregs and MDSCs during tumor progression (95). Within this microenvironmental architecture, MDSCs and Tregs demonstrate profound immunosuppressive synergy in both spatial colonization and functionality. The chemotactic network mediated by the CXCL12-CXCR4 signaling axis not only facilitates the initial localized enrichment of suppressive cells (96), but infiltrating MDSCs can also further leverage the CCL17/CCL22-CCR4 axis to specifically recruit Tregs into shared niches (97). At the level of metabolic regulation, these two suppressive populations can cooperatively utilize the CD39/CD73 metabolic pathway to generate copious amounts of adenosine, thereby dampening lymphocyte activity (98); in synergy, the massive accumulation of Tregs effectively sequesters free IL-2 within the microenvironment, triggering STAT5/TOX-mediated exhaustion programs within effector CD8+ T cells (96). Consequently, targeted blockade of key recruitment signals such as CXCR4 can effectively disrupt this spatial and metabolic synergistic network between MDSCs and Tregs, significantly reducing their joint infiltration and demonstrating tremendous therapeutic potential in restoring the anti-tumor functions of CD8+ T cells (96). Furthermore, given that the survival of CXCR6+ CD8+ T cells relies on CXCL16 secreted by IL1B+ M1-polarized macrophages, and that MDSCs and the tumor microenvironment exhibit a propensity to induce macrophage polarization toward M2-type tumor-associated macrophages (TAMs), this phenotypic shift in macrophage populations indirectly precipitates the loss of supportive molecules within the microenvironment and the ensuing exhaustion of effector T cells (99).

The intrinsic immunosuppressive state of MDSCs is not merely regulated by localized microenvironmental factors, but can also actively drive the adaptive evolution and therapeutic resistance of tumors via paracrine mechanisms. Prostaglandin E2 (PGE2) has been shown to potentially participate in amplifying the suppressive functions of MDSCs (100). Simultaneously, critical paracrine feedback loops exist between MDSCs and prostate cancer epithelial cells. In experimental models of mCRPC, PMN-MDSCs have been observed to secrete IL-23; via a paracrine route, this factor activates the STAT3-RORγ signaling network within tumor epithelial cells, subsequently linking to the upregulation of androgen receptor (AR) function. This phenomenon implies that myeloid cell activity within the microenvironment may constitute a vital component of the non-cell-autonomous regulatory mechanisms orchestrating the resistance phenotype against androgen deprivation therapy (ADT) in prostate cancer (101). These multidimensional intercellular communication networks illuminate that MDSCs exert intricate orchestrating and synergistic roles in the evolution of the prostate cancer immune microenvironment.

## 4 Intrinsic driving factors of tumor cells

Although the establishment of the canonical immune-”cold” phenotype in prostate cancer. is directly mediated by the suppressive cellular networks within the microenvironment, its core driving forces are fundamentally rooted in tumor-intrinsic genetic aberrations and epigenetic dysregulation. It is these intrinsic molecular derangements that initiate and perpetually dictate the remodeling trajectory of this immunosuppressive network. **Figure 3** provides a comprehensive overview of how tumor-intrinsic genomic mutations, adaptive responses to targeted therapies, and master epigenetic remodeling synergistically orchestrate the immunosuppressive prostate cancer microenvironment.

**Figure 3:**
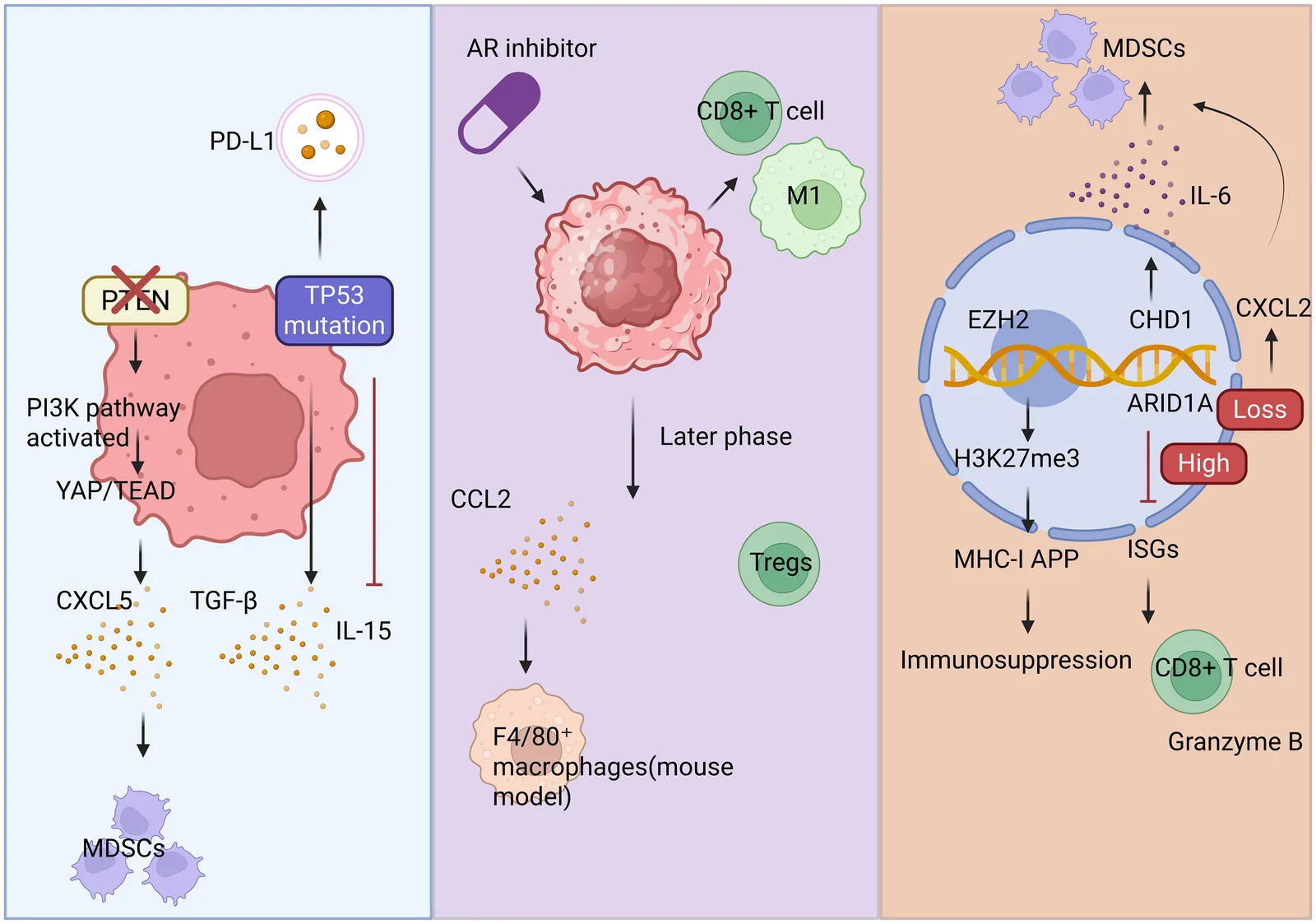
Tumor-intrinsic genomic, therapeutic, and epigenetic drivers of the immunosuppressive prostate cancer microenvironment. (Left) Genomic Aberrations: Tumor-intrinsic mutations actively orchestrate immune evasion. PTEN loss hyperactivates the PI3K-YAP/TEAD signaling cascade, upregulating CXCL5 secretion to specifically recruit myeloid-derived suppressor cells (MDSCs). Concomitantly, TP53 mutations promote the extracellular release of PD-L1 and TGF-β, while profoundly repressing the pro-inflammatory cytokine IL-15. (Middle) Therapeutic Dynamics: The biphasic microenvironmental response to androgen receptor (AR) inhibition. The initial therapeutic phase is characterized by the robust infiltration of anti-tumor CD8+ T cells and M1-polarized macrophages. However, during the later phase of adaptive resistance, tumor cells copiously secrete CCL2, driving the compensatory recruitment of F4/80+ macrophages and regulatory T cells (Tregs). (Right) Epigenetic Remodeling: Master epigenetic regulators intrinsically rewire immune visibility. EZH2 catalyzes H3K27me3 modification to transcriptionally silence the MHC-I antigen processing pathway (MHC-I APP). CHD1 drives IL-6 secretion to facilitate MDSC recruitment. Furthermore, ARID1A loss promotes CXCL2 expression and myeloid-cell recruitment, whereas high ARID1A expression has been reported to suppress interferon-stimulated genes (ISGs), culminating in the suppression of CD8+ T cell activity and Granzyme B production. PTEN, phosphatase and tensin homolog; PI3K, phosphoinositide 3-kinase; YAP, Yes-associated protein; TEAD, TEA domain transcription factor; MDSCs, myeloid-derived suppressor cells; PD-L1, programmed death-ligand 1; TGF-β, transforming growth factor-beta; IL, interleukin; AR, androgen receptor; Tregs, regulatory T cells; EZH2, enhancer of zeste homolog 2; MHC-I APP, major histocompatibility complex class I antigen processing pathway; CHD1, chromodomain helicase DNA binding protein 1; ARID1A, AT-rich interaction domain 1A; ISGs, interferon-stimulated genes.

### 4.1 PTEN inactivation

Phosphatase and tensin homolog (PTEN) on chromosome 10 is among the most frequently altered tumor suppressor genes in prostate cancer, exhibiting profound loss of function (102). Clinical and genomic profiling demonstrates that the incidence of PTEN deletion or mutation exhibits a pronounced escalating trajectory across the evolutionary continuum of prostate cancer progression. While PTEN inactivation is detected in approximately 20% of primary or localized prostate cancer specimens (102), this frequency profoundly surges to 40%–50% upon progression to the CRPC or mCRPC stage (103). Early microsatellite sequence analyses reported that the proportion of loss of heterozygosity (LOH) at the PTEN locus spanned between 10% and 55% across primary and advanced surgical cohorts (104, 105). In the more advanced CRPC setting, approximately 30% of patients may manifest deep, presumably homozygous, PTEN deletions, with a subset of cases concurrently harboring other genomic mutations and fusions (106).

The functional inactivation of PTEN is pervasively coupled with adverse clinicopathological hallmarks and dismal patient prognoses. PTEN status is recognized as an independent prognostic indicator for prostate cancer-specific mortality (107). A real-world retrospective cohort study indicated that among mCRPC patients undergoing clinical genomic profiling, PTEN loss-of-function statistically correlates with severely curtailed overall survival (108). Within the high-risk localized prostate cancer population, cases harboring PTEN deletions are more predisposed to presenting with advanced disease characteristics (e.g., a higher proportion of Stage IV disease) and are more frequently funneled into therapeutic paradigms of radiotherapy combined with androgen deprivation therapy (RT+ADT); in stark contrast, patients with intact PTEN more commonly undergo radical prostatectomy (109). Furthermore, up to 40% of PTEN-deficient primary tumors exhibit pronounced intratumoral heterogeneity, yet given the low rate of PTEN loss observed in isolated prostatic intraepithelial neoplasia (PIN), it is postulated that complete PTEN deletion is more likely a late-stage event during primary clonal evolution (110, 111). Regarding therapeutic response, PTEN deficiency is associated not only with resistance to chemotherapy and radiation (112), but murine models also reveal that under ADT, the inhibition of androgen signaling may accelerate the progression of PTEN-deficient tumors into invasive prostate cancer, underscoring an intricate reciprocal crosstalk between PTEN loss and AR pathway inhibition (113, 114).

At the cellular and molecular levels, PTEN acts as a phosphoinositide phosphatase whose primary physiological role is to dephosphorylate phosphatidylinositol-3,4,5-trisphosphate (PIP3) at the plasma membrane into phosphatidylinositol-4,5-bisphosphate (PIP2), thereby exerting critical negative regulation over the PI3K/AKT/mTOR signaling cascade (115). The hyperactivation of the PI3K survival signaling drives increased aerobic glycolysis within tumor cells. As outlined in Section 2.3, this metabolic shift can trigger elevated histone lactylation within the microenvironment, an epigenetic modification observed to correlate with compromised macrophage phagocytic activity and blunted anti-tumor immunity. Concurrently, the activation of mTOR is recognized to directly modulate inflammatory responses and immune cell differentiation within the TME (116). Beyond its canonical orchestration through the PI3K axis, PTEN also participates in disease progression by regulating cell polarity and growth signaling via non-canonical mechanisms, such as discrete protein-protein interactions (117).

One of the most salient hallmarks of PTEN inactivation-induced remodeling of the immune microenvironment is the robust chemotaxis and recruitment of MDSCs. In genetically engineered mouse models of prostate cancer harboring concurrent Pten and Smad4 deletions, researchers observed hyperactivation of YAP signaling; this cascade upregulates the secretion of the chemokine CXCL5 by tumor cells, directionally recruiting vast populations of MDSCs via the CXCR2 receptor axis (85). This finding has received preliminary biological corroboration in human prostate cancer specimens, where the expression of CXCL6 (the human ortholog of murine Cxcl5) is significantly upregulated in samples characterized by dense MDSC infiltration (85). Beyond the aberrant YAP-TEAD-CXCL5 axis, the IL-6 (88, 118) and JAK2/STAT3 cascades (119) have also been implicated in orchestrating this chemotactic influx. Furthermore, PTEN inactivation intricately correlates with the accelerated infiltration of TAMs and Tregs, further cementing a profoundly immunosuppressive landscape (120, 121).

### 4.2 Clinical association and molecular mechanism of immune regulation of TP53 mutation

TP53 mutations (mutp53) and its functional ablation in prostate cancer constitute one of the pivotal determinants driving dismal patient prognoses and the profound remodeling of the immune microenvironment. Epidemiological and cancer genomic profiling reveals that men harboring TP53 mutations exhibit a profoundly escalated risk of developing aggressive prostate cancer. Furthermore, elevated TP53 expression or its mutational status is clinically established as an independent prognostic indicator of adverse outcomes (122). At the molecular level, mutp53 proteins competitively bind to Src homology region 2 domain-containing phosphatase-1 (SHP1), thereby abrogating SHP1-mediated dephosphorylation and inhibition of STAT3 signaling (123). This competitive interaction perpetually sustains STAT3 in a constitutively phosphorylated and hyperactivated state.

In the context of evading host immune clearance, TP53 mutations not only perturb intracellular signaling cascades but also directly sculpt the surface antigen landscape and secretome of the tumor. On one hand, p53-deficient cells pervasively downregulate activating ligands for natural killer (NK) cells—such as NKG2D ligands, PVR, and Nectin-2—and simultaneously repress the cGAS-STING innate immune sensing pathway, fundamentally dampening their immunogenicity at the source (124, 125). On the other hand, because TP53 mutations frequently abrogate the expression of pro-apoptotic proteins including NOXA, BAX, and PUMA, tumor cells are endowed with formidable resistance against NK cell-mediated cytolytic eradication (126–128). TP53 mutations similarly exert profound, targeted suppression against adaptive immunity and macrophage populations. Mutated tumor cells unleash PD-L1-enriched extracellular vesicles into the microenvironment; by engaging PD-1, these vesicles not only directly precipitate T-cell exhaustion but also systemically abrogate the phagocytic capacity of macrophages (129). Within the paracrine network, these mutations orchestrate the profound repression of IL-15—a pro-inflammatory cytokine essential for activating CD8+ T and NK cells—while concomitantly triggering a massive surge in the secretion of the immunosuppressive mediator TGF-β (130, 131). This pronounced cytokine rewiring not only directly blunts the cytolytic activity of effector cells but also establishes an exquisitely permissive microenvironmental niche for the directional recruitment and polarization of suppressive populations, such as Tregs and PMN-MDSCs.

Notably, in exploring the intricate intersection of TP53 mutations, modalities of cell death, and the metabolic immune microenvironment, ferroptosis has emerged as a compelling potential regulatory mechanism. *In vitro* assays have corroborated that Erastin, a ferroptosis inducer, can trigger ferroptosis in prostate cancer cells by downregulating solute carrier family 7 member 11 (SLC7A11) and glutathione peroxidase 4 (GPX4)—a process that is not mechanistically absolute in its dependence on TP53 expression status. Nevertheless, cell lines expressing TP53 (e.g., LNCaP and DU145) exhibit profoundly higher baseline levels of intracellular reactive oxygen species (ROS) compared to TP53-deficient counterparts (e.g., PC3) (132). Given the pivotal role of ROS in orchestrating myeloid cell polarization and lymphocyte metabolism, this fundamental shift in baseline oxidative stress—dictated by disparate TP53 statuses—may serve as yet another formidable variable in the immunogenic rewiring of the tumor microenvironment.

### 4.3 The dual role of AR signal regulation in tumor control and immunosuppression

Blockade of the androgen receptor (AR) signaling pathway (e.g., via ADT or enzalutamide) serves not only as a canonical therapeutic modality to suppress prostate cancer proliferation but also as a pivotal trigger orchestrating the remodeling of the immune microenvironment. During the initial therapeutic window, AR inhibition can rapidly reverse the “immune-cold” phenotype of prostate cancer, igniting a multidimensional immune cascade. At the cellular level, the infiltration density of activated CD8+ T cells and M1-like TAMs surges precipitously; at the molecular level, the antigen-presenting capacity (MHC I/II) of tumor cells is significantly enhanced, concomitantly with the downregulation of phagocytosis-inhibitory signals (e.g., CD47) (133). Furthermore, accompanied by the massive release of damage-associated molecular patterns (DAMPs) and tumor antigens ensuing from target cell necrosis and apoptosis, this phase provides an exquisitely favorable sensitizing milieu for priming anti-tumor immunity (134). Nevertheless, this window of immune sensitization induced by endocrine therapy is remarkably transient, being rapidly counterbalanced by microenvironmental negative feedback mechanisms. As AR inhibition persists, the localized evolutionary trajectory of the tumor pivots from a pro-inflammatory state toward immune tolerance. Within the lymphoid compartment, the expansion of CD8+ effector T cells is invariably accompanied by the parallel proliferation of Tregs; this synchronous compensatory response directly abrogates the anti-tumor activity of effector cells (133). In PTEN-deficient prostate cancer models, around day 35 post-castration, as tumor cells begin to overcome endocrine blockade and resume growth, the expression of CCL2 within the microenvironment escalates precipitously (135). Concomitant with the copious secretion of CCL2, F4/80-positive macrophages cluster densely within the tumor bed, intimating that during the castration-adaptive phase, tumor cells can rewire the localized myeloid cell network via activation of the TNF-CCL2 signaling axis, thereby architecting a pro-tumorigenic stromal niche that sustains CRPC (135). This “control-and-suppress” duality orchestrated by AR signaling regulation partially elucidates the pervasive clinical dilemma confronting immune checkpoint blockade (ICB) therapies in prostate cancer practice.

### 4.4 Molecular network of epigenetic regulatory factors in prostate cancer immune escape

Among the myriad factors driving the lineage evolution and therapeutic resistance of prostate cancer, epigenetic remodeling is of paramount significance. Transcending canonical genomic mutations, epigenetic regulation—centered on histone modifications and chromatin conformational alterations—constitutes another crucial molecular foundation by which tumor cells evade host immune clearance. Recent studies indicate that key epigenetic regulators, such as EZH2 (histone methyltransferase), CHD1, and ARID1A (a core subunit of the SWI/SNF complex), are profoundly participating in and perpetuating the immunosuppressive microenvironment of prostate cancer through multidimensional signaling networks.

EZH2 serves as the pivotal catalytic core of PRC2, primarily responsible for catalyzing the trimethylation of lysine 27 on histone H3 (H3K27me3), thereby mediating the epigenetic silencing of target genes (136). Molecular mechanistic studies reveal that PRC2 mediates the coordinated transcriptional silencing of the MHC-I antigen processing pathway (MHC-I APP), facilitating the evasion of T-cell-mediated immunity (137). Research by Morel et al. demonstrates that beyond suppressing antigen presentation, EZH2 directly abrogates the innate immune sensing pathways of tumor cells. Investigations utilizing prostate cancer models and patient specimens corroborate that inhibiting the catalytic activity of EZH2 can lift the epigenetic silencing of genomic endogenous retroviral (ERV) sequences, prompting a pronounced cytosolic enrichment of endogenous double-stranded RNA (dsRNA). This “viral mimicry” effect specifically activates the STING-dependent innate immune signaling cascade. The activation of the STING pathway subsequently induces the transcription of the IFN-responsive gene network and upregulates the expression of chemokines that mediate effector T-cell infiltration (138). The aforementioned mechanisms indicate that releasing EZH2-mediated epigenetic repression can effectively restore the immunogenicity of prostate cancer. In preclinical experiments exploring the reversal of resistance to AR-targeted agents such as enzalutamide, EZH2 has similarly exhibited critical synergistic therapeutic potential. In subcutaneous xenograft and spontaneous prostate cancer mouse models, the combinatorial application of enzalutamide and the EZH2-specific inhibitor GSK-126 can significantly curb the growth of CRPC and reverse the tumor’s transdifferentiation trajectory toward a neuroendocrine phenotype (139). Compellingly, when this combinatorial intervention is implemented in immunodeficient mice, its anti-tumor efficacy is entirely abrogated; the success of this combinatorial regimen is fundamentally contingent upon the participation of an intact host immune system, specifically operating through the reawakening of the cytotoxicity of tumor-specific CD8+ T lymphocytes and the synthesis of IFN-γ (139). Furthermore, EZH2 exerts regulatory roles in reprogramming the glucose, fatty acid, and amino acid metabolic networks of prostate cancer cells, suggesting that it may indirectly support the immune evasion process through the microenvironmental redistribution of metabolites (140–142).

The high-frequency deletion of the CHD1 gene is also one of the common genomic mutational hallmarks in prostate cancer, occurring in approximately 8% of all cases (143). Pathologically, the loss of CHD1 is typically accompanied by defects in DNA repair mechanisms, aberrant genomic redistribution of AR, and alterations in transcriptional plasticity (118, 144, 145). However, utilizing genetic engineering to exclusively knock out the Chd1 gene in the murine prostate gland—despite observing glandular hyperplasia and prostatic intraepithelial neoplasia (PIN)—neither elicited significant alterations in cellular proliferation or survival nor was sufficient to induce invasive adenocarcinoma within a year. This suggests that CHD1 deletion alone is insufficient to serve as an initiating factor driving prostate malignancy (144). Distinct from the phenotype of isolated deletion, in PTEN-deficient models, the CHD1 protein remains stable and transforms into a pivotal epigenetic co-factor driving malignant progression, remodeling the tumor microenvironment, and mediating therapeutic resistance in prostate cancer (146). Functioning as a synthetic essential node under this genetic background, stabilized CHD1 recognizes and binds to the active histone mark H3K4me3 on chromatin, directly driving the sustained transcriptional activation of a pro-tumorigenic inflammatory hub—the TNFα/NF-κB signaling network (146). During tumor progression, IL-6 is one of the key target genes directly subjected to CHD1-mediated transcriptional regulation; the high concentrations of IL-6 secreted under CHD1’s drive not only promote tumor cell proliferation but also act as a crucial chemotactic signal, robustly recruiting and activating MDSCs to infiltrate the tumor tissue (88).

As a core subunit of the SWI/SNF chromatin remodeling complex, the downregulation or loss of ARID1A profoundly exacerbates immunosuppression in prostate cancer. This loss at the protein level is frequently driven by the microenvironment itself: localized chronic inflammatory signals can activate IKKβ, which directly phosphorylates ARID1A, prompting its degradation via the β-TRCP-dependent ubiquitin-proteasome pathway (10). The loss of ARID1A directly precipitates the enhancer inactivation and transcriptional silencing of the deubiquitinase A20, culminating in the robust transcription and secretion by tumor cells of chemotactic ligands for the CXCR2 receptor (such as CXCL2), thereby mediating the chemotaxis of MDSCs (10). Consequently, within the population of advanced prostate cancer patients harboring ARID1A functional defects, the combinatorial application of anti-NF-κB antibodies or CXCR2-specific inhibitors alongside immune checkpoint blockade therapy is recognized as a potential interventional strategy possessing a robust pathophysiological foundation (10).

Notably, high expression of ARID1A is equally capable of mediating tumor immune tolerance. Epigenomic studies confirm that elevated levels of ARID1A can drive localized chromatin compaction via phase separation mechanisms, spatially precluding the binding of transcriptional complexes and thereby broadly silencing the transcription of interferon-stimulated genes (ISGs) (147). The suppression of ISG signaling directly results in constrained local CD8+ T-cell infiltration and diminished granzyme B expression, rendering ARID1A-high prostate cancers primarily resistant to ICB therapy (147). Thus, it is evident that ARID1A is not a monofunctional transcriptional regulatory element, but rather a core epigenetic node deeply integrating chromatin conformational variations, inflammatory signal sensing, and immune cell chemotaxis. This dualistic effect intimates that the epigenetic regulation by ARID1A in prostate cancer manifests as a non-linear complex network, constituting a pivotal node mediating microenvironmental immunosuppression by directly coupling inflammatory signal sensing with chromatin conformational rearrangement.

## 5 Implications for treatment and future directions

### 5.1 Targeting immunosuppressive cell populations

The immunosuppressive properties within the prostate cancer microenvironment are perpetuated by a complex network synergistically architected by diverse cellular populations, including TAMs, MDSCs, Tregs, and CAFs. Therapeutic strategies aimed at dismantling this barrier can transcend the limitations of targeting a singular cell lineage by intervening across the following dimensions:

At the level of abrogating chemotactic recruitment and spatial exclusion barriers, severing the spatial migratory routes of suppressive cells into the tumor core represents a pivotal step in remodeling the microenvironment. For instance, the aberrantly high expression of CCL17/CCL22 by tumor and stromal cells specifically engages CCR4 to directionally recruit Treg infiltration; in a nonrandomized study of dogs with spontaneous advanced prostate cancer, mogamulizumab, an antibody against C-C motif chemokine receptor 4 (CCR4), combined with piroxicam depleted regulatory T cells from peripheral blood and tumor tissues and was associated with longer survival than piroxicam alone in matched control dogs (64). These findings require validation in human prostate cancer. Concurrently, the CXCR2 receptor axis orchestrates the massive chemotaxis of MDSCs. In head and neck squamous cell carcinoma, the utilization of CXCR2 antagonists (e.g., SX-682) has been proven to potently blockade MDSC infiltration, thereby relieving their suppression of CD8+ T cells (148). Given that the prostate cancer immune microenvironment is similarly highly reliant upon this signaling axis to recruit suppressive myeloid cells, CXCR2-targeted interventions manifest immense translational medical potential in combinatorial immunotherapies for prostate cancer. Furthermore, macrophages depend on the CXCL12-CXCR4 chemotactic axis to mediate the spatial colocalization of Tregs and CD8+ T cells, which subsequently triggers intense localized metabolic competition. Consequently, the combinatorial application of CXCR4-targeted inhibitors may effectively disrupt this suppressive spatial interactive network, holding promise for restoring the anti-tumor activity of effector T cells. Beyond blocking the spatial recruitment of suppressive cells, directly endowing effector T cells with the capacity to resist soluble suppressive factors within the tumor microenvironment constitutes another highly promising clinical interventional strategy. Prostate cancer canonically secretes TGF-β as a critical biochemical mechanism to suppress anti-tumor immunity and potentiate disease progression. In response to this cytokine-dominated immunosuppressive microenvironment, researchers have successfully leveraged genetic engineering to construct PSMA-targeted CAR-T cells co-expressing a dominant-negative TGF-β receptor II (dnTGF-βRII). Studies corroborate that this dominant-negative receptor effectively abrogates the TGF-β signaling cascade within T cells, thereby enabling CAR-T cells to exhibit significantly enhanced proliferative capacity, elevated cytokine secretion profiles, and formidable resistance against immune exhaustion. More crucially, in murine models of aggressive prostate cancer, CAR-T cells equipped with this receptor not only achieved long-term *in vivo* engraftment and survival but also successfully induced complete tumor eradication (149).

Additionally, reversing phenotypic plasticity and metabolic deprivation serves as another paradigm for circumventing prostate cancer immunosuppression. Targeting the metabolic hallmarks and phenotypic transitions of suppressive cells is critical for overcoming adaptive resistance. The SPP1+ TAM subpopulation, which is specifically enriched in prostate cancer, not only massively accumulates immunosuppressive adenosine within the microenvironment via high CD73 expression but also exhibits intrinsic resistance to conventional CSF-1R inhibitors. Its core mechanism of mediating immune resistance is profoundly contingent upon the adenosine signaling pathway; pharmacological blockade of the adenosine A2A receptor not only reverses the suppressive function of SPP1+ TAMs but also drastically sensitizes tumors to PD-1 blockade therapy *in vivo* (150). Furthermore, regarding CAFs—the principal components of the stroma—targeted inhibition of YAP1 can drive the transdifferentiation of pro-tumorigenic ECM-CAFs into Lym-CAFs endowed with anti-tumor immunomodulatory functions. This not only physically dismantles the constraints of the dense collagen matrix but also directly facilitates the deep infiltration of T cells via the release of chemokines (76).

### 5.2 Targeting intrinsic drivers of tumor cells

Relying exclusively on the blockade of the PD-1/PD-L1 pathway is insufficient to reverse the profound immunosuppressive network forged by chronic AR inhibition. However, given that conventional therapeutic modalities, such as ADT and radiotherapy (RT), exhibit distinct temporal orchestration in inducing tumor cell death, releasing immune signals, and triggering compensatory immune remodeling, future designs of combinatorial regimens integrating the AR axis with immunomodulators must prioritize the type of pharmacological intervention, dose intensity, treatment cycle, and, most crucially, the temporal sequencing of administration as core considerations. For instance, the administration of ADT prior to targeted radionuclide therapy (TRT) can maximally prime effector T cells; conversely, castration following radiotherapy triggers a compensatory recruitment of MDSCs, thereby generating antagonistic effects (90).

Furthermore, targeting the CHD1-IL-6-MDSC regulatory axis by combining IL-6 blockade with dual PD-1/CTLA-4 immune checkpoint inhibition has demonstrated pronounced synergistic anti-tumor efficacy in PTEN-deficient prostate cancer models (88). Concurrently, the blockade of the IL-6 pathway has been discovered to harbor the potential to uncouple anti-tumor immunity from autoimmune toxicity, thereby facilitating the mitigation of immune-related adverse events (irAEs) (151). Additionally, Mevrometostat, a targeted EZH2 inhibitor, has been developed, and emerging evidence indicates that the combinatorial application of Mevrometostat and enzalutamide in mCRPC yields significantly superior efficacy compared to enzalutamide monotherapy; nevertheless, its definitive efficacy and safety profiles await further validation in prospective clinical trials (152).

### 5.3 Patient stratification based on biomarkers

The profound molecular and spatial heterogeneity of mCRPC curtails the clinical benefit derived from conventional standard therapies, compelling biomarkers predicated on high-resolution liquid biopsy technologies to assume a pivotal role in patient stratification for targeted and immunotherapies. Regarding genomic hallmarks, circulating tumor DNA (ctDNA) has emerged as a core modality for non-invasive profiling, given its capacity to overcome the spatial limitations inherent to tissue biopsies. Quantifying microsatellite instability (MSI) status within ctDNA can effectively identify mCRPC patients who may derive clinical remission from immune checkpoint inhibitor therapy (153). Concurrently, ctDNA testing targeting alterations in the homologous recombination repair (HRR) pathway (e.g., BRCA1/2) constitutes the companion diagnostic rationale guiding the targeted application of PARP inhibitors (154).

Beyond genomic features, the systematic evaluation of peripheral blood soluble inflammatory mediators and immune cell fractions can serve as an objective surrogate reflecting the immune status of the tumor microenvironment. Current evidence indicates that aberrantly elevated levels of serum cytokines such as IL-6 correlate with the malignant progression of prostate cancer and resistance to endocrine therapy (155). Meanwhile, routine systemic inflammatory cell ratios, encompassing the neutrophil-to-lymphocyte ratio (NLR) and platelet-to-lymphocyte ratio (PLR), have proven capable of being integrated into clinical prognostic evaluation systems (156). High NLR and PLR states unmask the hyperactivity of systemic pro-tumorigenic inflammation alongside the relative exhaustion of anti-tumor immune effector mechanisms, thereby constituting independent predictors of dismal prognosis and immunotherapy resistance in mCRPC patients. Furthermore, B7-H3 (CD276) is an immune checkpoint molecule that exerts a core role in the immunosuppression, invasion, and metastasis of prostate cancer (157). A landmark translational medicine study published in 2025 successfully developed a novel liquid biopsy assay predicated on the quantitative measurement of B7-H3 protein expression within extracellular vesicles (EVs). By analyzing a massive cohort of prostate cancer patient plasma samples, this study revealed that as patients progressively and irreversibly deteriorate from the metastatic hormone-sensitive prostate cancer (mCSPC) stage to mCRPC, the titer of B7-H3-positive extracellular vesicles (B7-H3+ EVs) in their peripheral circulation exhibits a profoundly significant and precipitous elevation. More crucially for clinical guidance, survival analyses explicitly demonstrate that within the mCRPC cohort, the overall mortality risk for patients harboring a high abundance of B7-H3+ EVs escalates to more than twofold compared to those with low abundance (158). The aforementioned emerging biomarkers and their clinical implications are systematically summarized in **Table 1**.

**Table 1:** Emerging non-invasive biomarkers for immune microenvironment profiling and prognostic stratification in prostate cancer.

Biomarker category	Specific indicator	Sample source	Correlation with immune microenvironment and clinical prognosis
Peripheral Blood Inflammatory Markers	Neutrophil-to-lymphocyte ratio (NLR) & Platelet-to-lymphocyte ratio (PLR)	Peripheral blood	High NLR and PLR states reveal the activity of systemic pro-tumor inflammation and the relative exhaustion of anti-tumor immune effects, serving as independent predictors for poor prognosis and immunotherapy resistance in mCRPC patients.
Soluble Cytokines	Inflammatory mediators such as IL-6	Serum	The abnormal elevation of serum cytokines like IL-6 is directly associated with the malignant progression of prostate cancer and resistance to endocrine therapy.
Circulating Tumor DNA (ctDNA)	Microsatellite instability (MSI) status	Liquid biopsy (Blood)	Quantifying MSI in ctDNA can effectively identify mCRPC patients who may achieve clinical remission from immune checkpoint inhibitor therapy.
Circulating Tumor DNA (ctDNA)	Homologous recombination repair (HRR) pathway alterations (e.g., BRCA1/2)	Liquid biopsy (Blood)	The detection of HRR pathway variants in ctDNA serves as a companion diagnostic basis to guide the targeted application of PARP inhibitors.
Extracellular Vesicles (EVs)	B7-H3 positive extracellular vesicles (B7-H3+ EVs)	Plasma	The titer of B7-H3+ EVs in the peripheral circulation rises sharply as the disease progresses to mCRPC; patients with a high abundance of B7-H3+ EVs have more than twice the overall mortality risk compared to those with a low abundance.

This table systematically summarizes key liquid biopsy and peripheral blood biomarkers utilized in the clinical evaluation of advanced prostate cancer. The respective biomarker categories, specific molecular or cellular indicators, and biological sample sources are outlined alongside their implications for systemic immune microenvironment dynamics, treatment resistance, and overall clinical prognosis, particularly in the context of metastatic castration-resistant prostate cancer (mCRPC). Abbreviations: NLR, neutrophil-to-lymphocyte ratio; PLR, platelet-to-lymphocyte ratio; IL-6, interleukin-6; ctDNA, circulating tumor DNA; MSI, microsatellite instability; HRR, homologous recombination repair; PARP, poly (ADP-ribose) polymerase; EVs, extracellular vesicles; mCRPC, metastatic castration-resistant prostate cancer.

### 5.4 Therapeutic insights based on cutting-edge technologies

Recent clinical trials illustrate both the limitations and emerging directions of immunotherapy in prostate cancer (**Table 2**). Large randomized trials have not established a benefit from adding pembrolizumab or nivolumab to the evaluated hormonal or chemotherapy regimens in biomarker-unselected populations (159–162). Dual checkpoint blockade has demonstrated activity in a subset of patients, although comparative benefit remains unproven (163). Early-phase studies of antigen-directed cellular therapies and bispecific T-cell engagers have also reported antitumor activity, with toxicity and response durability requiring further evaluation (164, 165). These findings support further investigation of biomarker-guided patient selection and combinations informed by the tumor microenvironment. Ultimately, translating these microenvironmental insights into clinical practice means that, beyond conventional pharmacological blockade and endocrine-targeted therapies, future advances in prostate cancer immunotherapy will likely rely on the integration of interdisciplinary technologies. For instance, addressing the canonical immunosuppressive microenvironment of prostate cancer, the application of synthetic biology and “living therapeutics” manifests tremendous remodeling potential. The hypoxic cores pervasively characteristic of solid tumors, including prostate cancer, furnish an ideal colonization niche for non-pathogenic probiotics (e.g., Escherichia coli Nissle 1917) (166). More instructively, the prostate cancer microenvironment is frequently massively occupied by immunosuppressive populations such as MDSCs, which precipitate severe localized dual metabolic and immune suppression via the relentless depletion of L-arginine. Future investigations could explore the deployment of genetically engineered probiotics as vectors for localized metabolic interventions in prostate cancer. Upon targeted colonization within the tumor tissue, such microorganisms possess the capacity to constitutively convert the ammonia enriched within the microenvironment into L-arginine. This metabolic rewiring process directly antagonizes the localized amino acid deprivation effect in prostate cancer, thereby augmenting the infiltration extent and anti-tumor activity of effector T cells. The combinatorial application of these living microbial therapeutics with immune checkpoint inhibitors holds the promise of reversing the immunosuppressive state of prostate cancer and circumventing its intrinsic resistance, thereby providing a viable strategic paradigm for subsequent combinatorial therapeutic research.

**Table 2:** Selected recent clinical trials of immunotherapy in prostate cancer.

Trial/registration	Phase and population	Treatment	Main findings
KEYNOTE-921;NCT03834506	III;previously treated mCRPC;1,030 randomized patients	Pembrolizumab + docetaxel versus placebo + docetaxel	Neither primary endpoint, rPFS nor OS, met the prespecified criterion for statistical significance (159).
KEYNOTE-641;NCT03834493	III;chemotherapy-naive mCRPC;1,244 randomized patients	Pembrolizumab + enzalutamide versus placebo + enzalutamide	No improvement in OS or rPFS; treatment-related toxicity increased (160).
KEYNOTE-991;NCT04191096	III;mHSPC;1,251 randomized patients	Pembrolizumab versus placebo added to enzalutamide and androgen deprivation therapy	The rPFS endpoint was not met, and the trial was stopped for futility; severe adverse events were more frequent with pembrolizumab (161).
CheckMate 7DX;NCT04100018	III;mCRPC previously treated with androgen receptor pathway inhibitors;1,030 randomized patients	Nivolumab + docetaxel versus placebo + docetaxel	No improvement in rPFS or OS; grade 3–4 treatment-related adverse events were more frequent with nivolumab (162).
CheckMate 650;NCT02985957	II;chemotherapy-refractory mCRPC;259 randomized patients	Two nivolumab–ipilimumab regimens, ipilimumab alone, or cabazitaxel	Responses occurred in subsets of patients (163).
PSCA-directed CAR T-cell trial;NCT03873805	I;mCRPC;14 treated patients	PSCA-directed CAR T cells	PSA declines exceeding 30% occurred in 4/14 patients. Cystitis was dose-limiting, and CAR T-cell persistence was limited (164).
DeLLpro-300;NCT04702737	Ib;metastatic NEPC;40 enrolled patients	Tarlatamab, a DLL3-directed bispecific T-cell engager	Objective responses occurred in 4/38 evaluable patients, all with DLL3-positive tumors. Cytokine release syndrome was common (165).

This table summarizes selected clinical trials of immunotherapy in prostate cancer published between 2024 and 2026, including study phase, disease setting, sample size, treatment regimen, and principal efficacy and safety findings. Sample sizes refer to randomized, enrolled, or treated patients, as specified in each row; response denominators correspond to the populations evaluated in the original studies. Differences in patient selection, prior treatment, study design, and endpoints should be considered when interpreting the findings, which do not constitute direct comparisons across trials. Early-phase signals of antitumor activity should be distinguished from established comparative efficacy or survival benefit.

mCRPC, metastatic castration-resistant prostate cancer; mHSPC, metastatic hormone-sensitive prostate cancer; NEPC, neuroendocrine prostate cancer; rPFS, radiographic progression-free survival; OS, overall survival; PSCA, prostate stem cell antigen; CAR, chimeric antigen receptor; PSA, prostate-specific antigen; DLL3, delta-like ligand 3. Sample sizes refer to the populations specified in each row. Results from different trials should not be interpreted as direct comparisons.

## 6 Conclusion and outlook

The establishment of the immune-”cold” microenvironment in prostate cancer constitutes a multidimensional, multi-tiered, and complex evolutionary trajectory. This profoundly immune-excluded phenotype is driven not only by tumor-intrinsic determinants—such as PTEN inactivation, TP53 mutations, aberrant androgen receptor (AR) signaling, and epigenetic remodeling—but also heavily relies upon the spatial and biochemical suppressive networks synergistically architected by multicellular consortia within the microenvironment, including TAMs, Tregs, CAFs, and MDSCs. In recent years, the integrative application of single-cell transcriptomics and spatial multi-omics has elucidated the subpopulation heterogeneity and *in situ* communication wiring of this phenotype with high cellular precision.

Moving forward, research endeavors in prostate cancer immunotherapy must converge on the following pivotal directions: First, high-throughput multi-omics data must be further integrated to systematically dissect microenvironmental heterogeneity across distinct disease stages (e.g., mCRPC, NEPC, and DNPC) and disparate metastatic target organs, thereby comprehensively mapping the dynamic spatiotemporal landscape of immune evasion. Second, regarding clinical intervention strategies, dismantling microenvironmental barriers necessitates transcending singular immune checkpoint blockade to explore novel combinatorial regimens. These should target chemotactic recruitment networks (e.g., the CXCR2/CXCR4 axes), reverse phenotypic and metabolic plasticity, and abrogate epigenetic silencing (e.g., EZH2 targeting). Third, the implementation of precision medicine fundamentally relies upon high-resolution liquid biopsy technologies—such as ctDNA variant profiling and the quantitative detection of B7-H3+ extracellular vesicles—to achieve the precise stratification of patients receiving immunotherapy via novel biomarkers. Finally, the integration of cutting-edge interdisciplinary technologies (e.g., deploying genetically engineered probiotics to rewire the localized metabolic microenvironment) harbors the potential to fundamentally reverse the dual metabolic and immune suppressive state of the tumor bed. Sustained exploration across these dimensions will facilitate the dismantling of the spatial and metabolic exclusion barriers in prostate cancer, furnishing a robust theoretical foundation and practical guidance for overcoming clinical immunotherapy resistance.

## References

[1] Siegel RL Kratzer TB Giaquinto AN Sung H Jemal A . Cancer statistics, 2025. CA Cancer J Clin. (2025) 75:10–45. doi: 10.3322/caac.21871 39817679 PMC11745215

[2] Litwin MS Tan H-J . The diagnosis and treatment of prostate cancer: a review. JAMA. (2017) 317:2532–42. doi: 10.1001/jama.2017.7248 28655021

[3] Kirby M Hirst C Crawford ED . Characterising the castration-resistant prostate cancer population: a systematic review. Int J Clin Pract. (2011) 65:1180–92. doi: 10.1111/j.1742-1241.2011.02799.x 21995694

[4] Scher HI Solo K Valant J Todd MB Mehra M . Prevalence of prostate cancer clinical states and mortality in the United States: estimates using a dynamic progression model. PloS One. (2015) 10:e0139440. doi: 10.1371/journal.pone.0139440 26460686 PMC4603789

[5] Bilusic M Madan RA Gulley JL . Immunotherapy of prostate cancer: facts and hopes. Clin Cancer Res. (2017) 23:6764–70. doi: 10.1158/1078-0432.ccr-17-0019 28663235 PMC5690854

[6] Nicholson LT Fong L . Immune checkpoint inhibition in prostate cancer. Trends Cancer. (2020) 6:174–7. doi: 10.1016/j.trecan.2020.01.003 32101720

[7] Liu D Wang L Guo Y . Advances in and prospects of immunotherapy for prostate cancer. Cancer Lett. (2024) 601:217155. doi: 10.1016/j.canlet.2024.217155 39127338

[8] Stultz J Fong L . How to turn up the heat on the cold immune microenvironment of metastatic prostate cancer. Prostate Cancer Prostatic Dis. (2021) 24:697–717. doi: 10.1038/s41391-021-00340-5 33820953 PMC8384622

[9] Mirzaei S Paskeh MDA Okina E Gholami MH Hushmandi K Hashemi M . Molecular landscape of lncRNAs in prostate cancer: a focus on pathways and therapeutic targets for intervention. J Exp Clin Cancer Res. (2022) 41:214. doi: 10.1186/s13046-022-02406-1 35773731 PMC9248128

[10] Li N Liu Q Han Y Pei S Cheng B Xu J . ARID1A loss induces polymorphonuclear myeloid-derived suppressor cell chemotaxis and promotes prostate cancer progression. Nat Commun. (2022) 13:7281. doi: 10.1038/s41467-022-34871-9 36435834 PMC9701216

[11] Davies AH Beltran H Zoubeidi A . Cellular plasticity and the neuroendocrine phenotype in prostate cancer. Nat Rev Urol. (2018) 15:271–86. doi: 10.1038/nrurol.2018.22 29460922

[12] Bian X Wang W Abudurexiti M Zhang X Ma W Shi G . Integration analysis of single-cell multi-omics reveals prostate cancer heterogeneity. Adv Sci (Weinh). (2024) 11:e2305724. doi: 10.1002/advs.202305724 38483933 PMC11095148

[13] Hatano K Fujita K Nonomura N . Application of anti-inflammatory agents in prostate cancer. J Clin Med. (2020) 9:2680. doi: 10.3390/jcm9082680 32824865 PMC7464558

[14] Jafari S Molavi O Kahroba H Hejazi MS Maleki-Dizaji N Barghi S . Clinical application of immune checkpoints in targeted immunotherapy of prostate cancer. Cell Mol Life Sci. (2020) 77:3693–710. doi: 10.1007/s00018-020-03459-1 32006051 PMC11104895

[15] Feng D-C Zhu W-Z Wang J Li D-X Shi X Xiong Q . The implications of single-cell RNA-seq analysis in prostate cancer: unraveling tumor heterogeneity, therapeutic implications and pathways towards personalized therapy. Mil Med Res. (2024) 11:21. doi: 10.1186/s40779-024-00526-7 38605399 PMC11007901

[16] Yu H Wang Y He M Chen Y Bi J Lin X . Integrated single-cell and spatial transcriptomic profiling decodes lineage plasticity and immune microenvironment remodeling in prostate cancer progression. Mol Cancer. (2026) 25:88. doi: 10.1186/s12943-026-02617-6 41764551 PMC13049746

[17] Mei S Zhang H Hirz T Jeffries NE Xu Y Baryawno N . Single-cell and spatial transcriptomics reveal a tumor-associated macrophage subpopulation that mediates prostate cancer progression and metastasis. Mol Cancer Res. (2025) 23:653–65. doi: 10.1158/1541-7786.mcr-24-0791 40105746 PMC12221797

[18] Graham MK Wang R Chikarmane R Abel B Vaghasia A Gupta A . Convergent alterations in the tumor microenvironment of MYC-driven human and murine prostate cancer. Nat Commun. (2024) 15:7414. doi: 10.1038/s41467-024-51450-2 39198404 PMC11358296

[19] Kiviaho A Eerola SK Kallio HML Andersen MK Hoikka M Tiihonen AM . Single cell and spatial transcriptomics highlight the interaction of club-like cells with immunosuppressive myeloid cells in prostate cancer. Nat Commun. (2024) 15:9949. doi: 10.1038/s41467-024-54364-1 39550375 PMC11569175

[20] Takahara T Taniguchi N Sassa N Tsuzuki T . Spatial transcriptomics of intraductal carcinoma of the prostate. Histopathology. (2025) 87:745–56. doi: 10.1111/his.15551 40964799

[21] Denisenko E Guo BB Jones M Hou R de Kock L Lassmann T . Systematic assessment of tissue dissociation and storage biases in single-cell and single-nucleus RNA-seq workflows. Genome Biol. (2020) 21:130. doi: 10.1186/s13059-020-02048-6 32487174 PMC7265231

[22] You Y Fu Y Li L Zhang Z Jia S Lu S . Systematic comparison of sequencing-based spatial transcriptomic methods. Nat Methods. (2024) 21:1743–54. doi: 10.1038/s41592-024-02325-3 38965443 PMC11399101

[23] Luecken MD Buttner M Chaichoompu K Danese A Interlandi M Mueller MF . Benchmarking atlas-level data integration in single-cell genomics. Nat Methods. (2022) 19:41–50. doi: 10.1038/s41592-021-01336-8 PMC8748196 34949812

[24] Hirz T Mei S Sarkar H Kfoury Y Wu S Verhoeven BM . Dissecting the immune suppressive human prostate tumor microenvironment via integrated single-cell and spatial transcriptomic analyses. Nat Commun. (2023) 14:663. doi: 10.1038/s41467-023-36325-2 36750562 PMC9905093

[25] Apostolov E Roden DL Holliday H Cazet A Harvey K Zhang H . Single-cell and spatial transcriptomic profiling reveals epithelial functional states and fibroblast phenotypes in hormone therapy-naïve localized prostate cancer. Cancer Res. (2026) 86:1836–53. doi: 10.1158/0008-5472.can-25-1202 41879555

[26] Tuong ZK Loudon KW Berry B Richoz N Jones J Tan X . Resolving the immune landscape of human prostate at a single-cell level in health and cancer. Cell Rep. (2021) 37:110132. doi: 10.1016/j.celrep.2021.110132 34936871 PMC8721283

[27] Jiao S Subudhi SK Aparicio A Ge Z Guan B Miura Y . Differences in tumor microenvironment dictate T helper lineage polarization and response to immune checkpoint therapy. Cell. (2019) 179:1177–90.e13. doi: 10.1016/j.cell.2019.10.029 31730856

[28] Kfoury Y Baryawno N Severe N Mei S Gustafsson K Hirz T . Human prostate cancer bone metastases have an actionable immunosuppressive microenvironment. Cancer Cell. (2021) 39:1464–78 e8. doi: 10.1016/j.ccell.2021.09.005 34719426 PMC8578470

[29] Zhang Q Liu S Zhang Q Xiong Z Wang AR Myers L . Interleukin-17 promotes development of castration-resistant prostate cancer potentially through creating an immunotolerant and pro-angiogenic tumor microenvironment. Prostate. (2014) 74:869–79. doi: 10.1002/pros.22805 24691769 PMC4063299

[30] Lan R Zhang K Niu T You Z . Genetic alterations of interleukin-17 and related genes in human prostate cancer. Am J Clin Exp Urol. (2019) 7:352–77. PMC6971475 31970232

[31] Zhang Q Liu S Parajuli KR Zhang W Zhang K Mo Z . Interleukin-17 promotes prostate cancer via MMP7-induced epithelial-to-mesenchymal transition. Oncogene. (2017) 36:687–99. doi: 10.1038/onc.2016.240 PMC5213194 27375020

[32] Batlle E Massague J . Transforming growth factor-beta signaling in immunity and cancer. Immunity. (2019) 50:924–40. doi: 10.1016/j.immuni.2019.03.024 30995507 PMC7507121

[33] Herroon MK Rajagurubandara E Rudy DL Chalasani A Hardaway AL Podgorski I . Macrophage cathepsin K promotes prostate tumor progression in bone. Oncogene. (2013) 32:1580–93. doi: 10.1038/onc.2012.166 PMC3913739 22614014

[34] Mizutani K Sud S McGregor NA Martinovski G Rice BT Craig MJ . The chemokine CCL2 increases prostate tumor growth and bone metastasis through macrophage and osteoclast recruitment. Neoplasia. (2009) 11:1235–42. doi: 10.1593/neo.09988 19881959 PMC2767225

[35] Lu X Horner JW Paul E Shang X Troncoso P Deng P . Effective combinatorial immunotherapy for castration-resistant prostate cancer. Nature. (2017) 543:728–32. doi: 10.1038/nature21676 28321130 PMC5374023

[36] Cook LM Shay G Araujo A Lynch CC . Integrating new discoveries into the "vicious cycle" paradigm of prostate to bone metastases. Cancer Metastasis Rev. (2014) 33:511–25. doi: 10.1007/s10555-014-9494-4 24414228 PMC4096318

[37] Bhinder B Ferguson A Sigouros M Uppal M Elsaeed AG Bareja R . Immunogenomic landscape of neuroendocrine prostate cancer. Clin Cancer Res. (2023) 29:2933–43. doi: 10.1158/1078-0432.ccr-22-3743 37223924 PMC10524949

[38] Wang Y Wang Y Ci X Choi SYC Crea F Lin D . Molecular events in neuroendocrine prostate cancer development. Nat Rev Urol. (2021) 18:581–96. doi: 10.1038/s41585-021-00490-0 34290447 PMC10802813

[39] Liu K Zhang Y Du G Chen X Xiao L Jiang L . 5-HT orchestrates histone serotonylation and citrullination to drive neutrophil extracellular traps and liver metastasis. J Clin Invest. (2025) 135:e183544. doi: 10.1172/jci183544 39903533 PMC11996869

[40] Chen X Bao W Liu K Jing N Du G Jiang L . O-GalNAc glycosylation activates MBL-mediated complement and coagulation cascades to drive organotropic metastasis. Adv Sci (Weinh). (2025) 12:e04809. doi: 10.1002/advs.202504809 40492591 PMC12407318

[41] Davies A Zoubeidi A Selth LA . The epigenetic and transcriptional landscape of neuroendocrine prostate cancer. Endocr Relat Cancer. (2020) 27:R35–50. doi: 10.1530/erc-19-0420 31804971

[42] Xiong X Xie J Dai P Cheng C Liu J Huang G . Integrative bulk and single-cell transcriptomics link EZH2 to immunosuppressive programs and tumor-Treg crosstalk in castration-resistant prostate cancer. Front Immunol. (2026) 17:1725097. doi: 10.3389/fimmu.2026.1725097 41710887 PMC12909565

[43] Labrecque MP Coleman IM Brown LG True LD Kollath L Lakely B . Molecular profiling stratifies diverse phenotypes of treatment-refractory metastatic castration-resistant prostate cancer. J Clin Invest. (2019) 129:4492–505. doi: 10.1172/jci128212 31361600 PMC6763249

[44] Vellky JE Ricke WA . Development and prevalence of castration-resistant prostate cancer subtypes. Neoplasia. (2020) 22:566–75. doi: 10.1016/j.neo.2020.09.002 32980775 PMC7522286

[45] Tang F Xu D Wang S Wong CK Martinez-Fundichely A Lee CJ . Chromatin profiles classify castration-resistant prostate cancers suggesting therapeutic targets. Science. (2022) 376:eabe1505. doi: 10.1126/science.abe1505 35617398 PMC9299269

[46] Pitzen SP Rudenick AN Qiu Y Zhang W Munro SA McCluskey BM . Comparative transcriptomics reveals a mixed basal, club, and hillock epithelial cell identity in castration-resistant prostate cancer. Proc Natl Acad Sci USA. (2025) 122:e2415308122. doi: 10.1073/pnas.2415308122 39913208 PMC11831193

[47] Wang J Wu R Tuo Z Okoli UA Wang Z Chaipanichkul P . Deciphering double-negative prostate cancer: from aggressive subtype to novel therapeutic paradigms. Cell Commun Signal. (2026) 24:264. doi: 10.1186/s12964-026-02795-2 41877195 PMC13137589

[48] Kamijima T Izumi K Hiratsuka K Inaba T Koketsu Y Nakagawa R . Fibroblast-mediated KRAS activation in double-negative prostate cancer. Cell Death Dis. (2026) 17:403. doi: 10.1038/s41419-026-08800-3 42069672 PMC13139504

[49] Wise DR Schneider JA Armenia J Febles VA McLaughlin B Brennan R . Dickkopf-1 can lead to immune evasion in metastatic castration-resistant prostate cancer. JCO Precis Oncol. (2020) 4:PO.20.00097. doi: 10.1200/po.20.00097 33015525 PMC7529521

[50] Yang H Zhang Q Xu M Wang L Chen X Feng Y . CCL2-CCR2 axis recruits tumor associated macrophages to induce immune evasion through PD-1 signaling in esophageal carcinogenesis. Mol Cancer. (2020) 19:41. doi: 10.1186/s12943-020-01165-x 32103760 PMC7045401

[51] Escamilla J Schokrpur S Liu C Priceman SJ Moughon D Jiang Z . CSF1 receptor targeting in prostate cancer reverses macrophage-mediated resistance to androgen blockade therapy. Cancer Res. (2015) 75:950–62. doi: 10.1158/0008-5472.can-14-0992 25736687 PMC4359956

[52] DeNardo DG Ruffell B . Macrophages as regulators of tumour immunity and immunotherapy. Nat Rev Immunol. (2019) 19:369–82. doi: 10.1038/s41577-019-0127-6 30718830 PMC7339861

[53] Kurahara H Takao S Kuwahata T Nagai T Ding Q Maeda K . Clinical significance of folate receptor beta-expressing tumor-associated macrophages in pancreatic cancer. Ann Surg Oncol. (2012) 19:2264–71. doi: 10.1245/s10434-012-2263-0 22350599

[54] Sharma A Seow JJW Dutertre CA Pai R Bleriot C Mishra A . Onco-fetal reprogramming of endothelial cells drives immunosuppressive macrophages in hepatocellular carcinoma. Cell. (2020) 183:377–94:e21. doi: 10.1016/j.cell.2020.08.040 32976798

[55] Nalio Ramos R Missolo-Koussou Y Gerber-Ferder Y Bromley CP Bugatti M Nunez NG . Tissue-resident FOLR2(+) macrophages associate with CD8(+) T cell infiltration in human breast cancer. Cell. (2022) 185:1189–207:e25. doi: 10.1016/j.cell.2022.02.021 35325594

[56] Bao X Li Q Chen D Dai X Liu C Tian W . A multiomics analysis-assisted deep learning model identifies a macrophage-oriented module as a potential therapeutic target in colorectal cancer. Cell Rep Med. (2024) 5:101399. doi: 10.1016/j.xcrm.2024.101399 38307032 PMC10897549

[57] Cresswell GM Wang B Kischuk EM Broman MM Alfar RA Vickman RE . Folate receptor beta designates immunosuppressive tumor-associated myeloid cells that can be reprogrammed with folate-targeted drugs. Cancer Res. (2021) 81:671–84. doi: 10.1158/0008-5472.can-20-1414 33203700 PMC10987201

[58] Jiang D Xiao R Bai J Wang X Fang Y Liu J . FOLR2(+) macrophages in cancer: allies or enemies. Cell Commun Signal. (2025) 23:261. doi: 10.1186/s12964-025-02257-1 40457348 PMC12131471

[59] Trizzino M Zucco A Deliard S Wang F Barbieri E Veglia F . EGR1 is a gatekeeper of inflammatory enhancers in human macrophages. Sci Adv. (2021) 7:eaaz8836. doi: 10.1126/sciadv.aaz8836 33523892 PMC7806227

[60] Yoshimura T . The chemokine MCP-1 (CCL2) in the host interaction with cancer: a foe or ally? Cell Mol Immunol. (2018) 15:335–45. doi: 10.1038/cmi.2017.135 29375123 PMC6052833

[61] Lin L Chen YS Yao YD Chen JQ Chen JN Huang SY . CCL18 from tumor-associated macrophages promotes angiogenesis in breast cancer. Oncotarget. (2015) 6:34758–73. doi: 10.18632/oncotarget.5325 26416449 PMC4741488

[62] Chen J Yao Y Gong C Yu F Su S Chen J . CCL18 from tumor-associated macrophages promotes breast cancer metastasis via PITPNM3. Cancer Cell. (2011) 19:541–55. doi: 10.1016/j.ccr.2011.02.006 21481794 PMC3107500

[63] Lian C Liu L Zhang X . Targeting the chemokine-Treg axes in tumor immune evasion: from mechanisms to therapeutic opportunities. Front Immunol. (2026) 17:1779388. doi: 10.3389/fimmu.2026.1779388 41890756 PMC13013285

[64] Maeda S Motegi T Iio A Kaji K Goto-Koshino Y Eto S . Anti-CCR4 treatment depletes regulatory T cells and leads to clinical activity in a canine model of advanced prostate cancer. J Immunother Cancer. (2022) 10:e003731. doi: 10.1136/jitc-2021-003731 35131860 PMC8804701

[65] Li J Zhang L Liu R Xu C Tang H Zou Y . CXCL12/CXCR4 axis governs Treg spatial dominance over CD8+ T cells via IL-2 sequestration: a dual therapeutic target in prostate cancer. Front Immunol. (2025) 16:1626708. doi: 10.3389/fimmu.2025.1626708 40698080 PMC12279891

[66] Phillips D Matusiak M Gutierrez BR Bhate SS Barlow GL Jiang S . Immune cell topography predicts response to PD-1 blockade in cutaneous T cell lymphoma. Nat Commun. (2021) 12:6726. doi: 10.1038/s41467-021-26974-6 34795254 PMC8602403

[67] Atmakuri A Wala J Amiryousefi A Lin J-R Labadie B Chaudagar K . 773 spatial characterization of the immune microenvironment of localized prostate cancer reveals an immunogenic subset of high-risk tumors. J Immunother Cancer. (2025) 13. doi: 10.1136/jitc-2025-sitc2025.0773

[68] Ak C Sayar Z Thibault G Burlingame EA Kuykendall MJ Eng J . Multiplex imaging of localized prostate tumors reveals altered spatial organization of AR-positive cells in the microenvironment. iScience. (2024) 27:110668. doi: 10.1016/j.isci.2024.110668 39246442 PMC11379676

[69] Sugimoto Y Hirota M Yoshikawa K Sumitomo M Nakamura K Ueda R . The therapeutic potential of a novel PSMA antibody and its IL-2 conjugate in prostate cancer. Anticancer Res. (2014) 34:89–97. 24403448

[70] Sim GC Martin-Orozco N Jin L Yang Y Wu S Washington E . IL-2 therapy promotes suppressive ICOS+ Treg expansion in melanoma patients. J Clin Invest. (2014) 124:99–110. doi: 10.1172/JCI46266 PMC3871216 24292706

[71] Yan Y Huang L Liu Y Yi M Chu Q Jiao D . Metabolic profiles of regulatory T cells and their adaptations to the tumor microenvironment: implications for antitumor immunity. J Hematol Oncol. (2022) 15:104. doi: 10.1186/s13045-022-01322-3 35948909 PMC9364625

[72] von Amsberg G Alsdorf W Karagiannis P Coym A Kaune M Werner S . Immunotherapy in advanced prostate cancer-light at the end of the tunnel? Int J Mol Sci. (2022) 23:2569. doi: 10.3390/ijms23052569 35269712 PMC8910587

[73] LeBleu VS Kalluri R . A peek into cancer-associated fibroblasts: origins, functions and translational impact. Dis Model Mech. (2018) 11:dmm029447. doi: 10.1242/dmm.029447 29686035 PMC5963854

[74] Wu Y Xu D Shi R Zhan M Xu S Wang X . Cancer-associated fibroblasts in prostate cancer: unraveling mechanisms and therapeutic implications. Oncol Res. (2026) 34:10. doi: 10.32604/or.2025.073265 41613792 PMC12848666

[75] Yang FC Chen S Clegg T Li X Morgan T Estwick SA . Nf1+/- mast cells induce neurofibroma like phenotypes through secreted TGF-beta signaling. Hum Mol Genet. (2006) 15:2421–37. doi: 10.1093/hmg/ddl165 16835260 PMC3024714

[76] Song H Lu T Han D Zhang J Gan L Xu C . YAP1 inhibition induces phenotype switching of cancer-associated fibroblasts to tumor suppressive in prostate cancer. Cancer Res. (2024) 84:3728–42. doi: 10.1158/0008-5472.can-24-0932 39137404 PMC11565174

[77] Fiaschi T Marini A Giannoni E Taddei ML Gandellini P De Donatis A . Reciprocal metabolic reprogramming through lactate shuttle coordinately influences tumor-stroma interplay. Cancer Res. (2012) 72:5130–40. doi: 10.1158/0008-5472.can-12-1949 22850421

[78] Haas R Smith J Rocher-Ros V Nadkarni S Montero-Melendez T D'Acquisto F . Lactate regulates metabolic and pro-inflammatory circuits in control of T cell migration and effector functions. PloS Biol. (2015) 13:e1002202. doi: 10.1371/journal.pbio.1002202 26181372 PMC4504715

[79] Hu G Cheng P Pan J Wang S Ding Q Jiang Z . An IL6-adenosine positive feedback loop between CD73(+) gammadeltaTregs and CAFs promotes tumor progression in human breast cancer. Cancer Immunol Res. (2020) 8:1273–86. doi: 10.1158/2326-6066.CIR-19-0923 32847938

[80] Kalluri R . The biology and function of fibroblasts in cancer. Nat Rev Cancer. (2016) 16:582–98. doi: 10.1038/nrc.2016.73 27550820

[81] Feig C Jones JO Kraman M Wells RJ Deonarine A Chan DS . Targeting CXCL12 from FAP-expressing carcinoma-associated fibroblasts synergizes with anti-PD-L1 immunotherapy in pancreatic cancer. Proc Natl Acad Sci USA. (2013) 110:20212–7. doi: 10.1073/pnas.1320318110 24277834 PMC3864274

[82] Yang X Lin Y Shi Y Li B Liu W Yin W . FAP promotes immunosuppression by cancer-associated fibroblasts in the tumor microenvironment via STAT3-CCL2 signaling. Cancer Res. (2016) 76:4124–35. doi: 10.1158/0008-5472.can-15-2973 27216177

[83] Sieminska I Baran J . Myeloid-derived suppressor cells as key players and promising therapy targets in prostate cancer. Front Oncol. (2022) 12:862416. doi: 10.3389/fonc.2022.862416 PMC9289201 35860573

[84] Calcinotto A Spataro C Zagato E Di Mitri D Gil V Crespo M . IL-23 secreted by myeloid cells drives castration-resistant prostate cancer. Nature. (2018) 559:363–9. doi: 10.1038/s41586-018-0266-0 29950727 PMC6461206

[85] Wang G Lu X Dey P Deng P Wu CC Jiang S . Targeting YAP-dependent MDSC infiltration impairs tumor progression. Cancer Discov. (2016) 6:80–95. doi: 10.1158/2159-8290.cd-15-0224 26701088 PMC4707102

[86] Garcia AJ Ruscetti M Arenzana TL Tran LM Bianci-Frias D Sybert E . Pten null prostate epithelium promotes localized myeloid-derived suppressor cell expansion and immune suppression during tumor initiation and progression. Mol Cell Biol. (2014) 34:2017–28. doi: 10.1128/mcb.00090-14 24662052 PMC4019050

[87] Burkhardt L Fuchs S Krohn A Masser S Mader M Kluth M . CHD1 is a 5q21 tumor suppressor required for ERG rearrangement in prostate cancer. Cancer Res. (2013) 73:2795–805. doi: 10.1158/0008-5472.can-12-1342 23492366

[88] Zhao D Cai L Lu X Liang X Li J Chen P . Chromatin regulator CHD1 remodels the immunosuppressive tumor microenvironment in PTEN-deficient prostate cancer. Cancer Discov. (2020) 10:1374–87. doi: 10.1158/2159-8290.cd-19-1352 32385075 PMC7483306

[89] Jayaprakash P Ai M Liu A Budhani P Bartkowiak T Sheng J . Targeted hypoxia reduction restores T cell infiltration and sensitizes prostate cancer to immunotherapy. J Clin Invest. (2018) 128:5137–49. doi: 10.1172/jci96268 30188869 PMC6205399

[90] Muralidhar A Hernandez R Morris ZS Comas Rojas H Bio Idrissou M Weichert JP . Myeloid-derived suppressor cells attenuate the antitumor efficacy of radiopharmaceutical therapy using (90)Y-NM600 in combination with androgen deprivation therapy in murine prostate tumors. J Immunother Cancer. (2024) 12:e008760. doi: 10.1136/jitc-2023-008760 38663936 PMC11043705

[91] Cheng Y Tang XY Li YX Zhao DD Cao QH Wu HX . Depression-induced neuropeptide Y secretion promotes prostate cancer growth by recruiting myeloid cells. Clin Cancer Res. (2019) 25:2621–32. doi: 10.1158/1078-0432.ccr-18-2912 30504424

[92] Bronte V Brandau S Chen SH Colombo MP Frey AB Greten TF . Recommendations for myeloid-derived suppressor cell nomenclature and characterization standards. Nat Commun. (2016) 7:12150. doi: 10.1038/ncomms12150 27381735 PMC4935811

[93] Saligan LN Lukkahatai N Zhang ZJ Cheung CW Wang XM . Altered Cd8+ T lymphocyte response triggered by arginase 1: implication for fatigue intensification during localized radiation therapy in prostate cancer patients. Neuropsychiatry (London). (2018) 8:1249–62. doi: 10.4172/neuropsychiatry.1000454 30364895 PMC6198670

[94] Hossain DM Pal SK Moreira D Duttagupta P Zhang Q Won H . TLR9-targeted STAT3 silencing abrogates immunosuppressive activity of myeloid-derived suppressor cells from prostate cancer patients. Clin Cancer Res. (2015) 21:3771–82. doi: 10.1158/1078-0432.ccr-14-3145 25967142 PMC4537814

[95] Jachetti E Cancila V Rigoni A Bongiovanni L Cappetti B Belmonte B . Cross-talk between myeloid-derived suppressor cells and mast cells mediates tumor-specific immunosuppression in prostate cancer. Cancer Immunol Res. (2018) 6:552–65. doi: 10.1158/2326-6066.cir-17-0385 29523597

[96] Huang J Ojo A Tsao S Horowitz A Kyprianou N Tsao CK . Overcoming immune evasion in the prostate tumor microenvironment: novel targeted strategies to improve treatment outcomes. Cancers (Basel). (2025) 17:3441. doi: 10.3390/cancers17213441 41228234 PMC12609306

[97] Schlecker E Stojanovic A Eisen C Quack C Falk CS Umansky V . Tumor-infiltrating monocytic myeloid-derived suppressor cells mediate CCR5-dependent recruitment of regulatory T cells favoring tumor growth. J Immunol. (2012) 189:5602–11. doi: 10.4049/jimmunol.1201018 23152559

[98] Allard B Allard D Buisseret L Stagg J . The adenosine pathway in immuno-oncology. Nat Rev Clin Oncol. (2020) 17:611–29. doi: 10.1038/s41571-020-0382-2 32514148

[99] Xingming Z Wang J Abudurexiti M Du L Zhang W Ni X . Dual regulation of CXCR6(+)CD8(+) T cells modulates cytotoxic and exhaustion-associated programs during prostate cancer progression. J Immunother Cancer. (2026) 14:e014276. doi: 10.1136/jitc-2025-014276 41819547 PMC12983932

[100] Tomic S Joksimovic B Bekic M Vasiljevic M Milanovic M Colic M . Prostaglanin-E2 potentiates the suppressive functions of human mononuclear myeloid-derived suppressor cells and increases their capacity to expand IL-10-producing regulatory T cell subsets. Front Immunol. (2019) 10:475. doi: 10.3389/fimmu.2019.00475 PMC6431635 30936876

[101] Khaki Bakhtiarvand V Ramezani-Ali Akbari K Amir Jalali S Hojjat-Farsangi M Jeddi-Tehrani M Shokri F . Myeloid-derived suppressor cells (MDSCs) depletion by cabozantinib improves the efficacy of anti-HER2 antibody-based immunotherapy in a 4T1-HER2 murine breast cancer model. Int Immunopharmacol. (2022) 113:109470. doi: 10.1016/j.intimp.2022.109470 36435059

[102] Jamaspishvili T Berman DM Ross AE Scher HI De Marzo AM Squire JA . Clinical implications of PTEN loss in prostate cancer. Nat Rev Urol. (2018) 15:222–34. doi: 10.1038/nrurol.2018.9 29460925 PMC7472658

[103] Robinson D Van Allen EM Wu YM Schultz N Lonigro RJ Mosquera JM . Integrative clinical genomics of advanced prostate cancer. Cell. (2015) 161:1215–28. doi: 10.1016/j.cell.2015.05.001 26000489 PMC4484602

[104] Cairns P Okami K Halachmi S Halachmi N Esteller M Herman JG . Frequent inactivation of PTEN/MMAC1 in primary prostate cancer. Cancer Res. (1997) 57:4997–5000. 9371490

[105] Suzuki H Freije D Nusskern DR Okami K Cairns P Sidransky D . Interfocal heterogeneity of PTEN/MMAC1 gene alterations in multiple metastatic prostate cancer tissues. Cancer Res. (1998) 58:204–9. 9443392

[106] Esteban-Villarrubia J Ballesteros PA Martín-Serrano M Vico MR Funes JM de Velasco G . Mechanisms of immune evasion in PTEN loss prostate cancer. Immuno [Internet]. (2024) 4:444–60. doi: 10.3390/immuno404002830654563

[107] de Bono J Mateo J Fizazi K Saad F Shore N Sandhu S . Olaparib for metastatic castration-resistant prostate cancer. N Engl J Med. (2020) 382:2091–102. doi: 10.1056/nejmoa1911440 32343890

[108] Gupta S To TM Graf R Kadel EE 3rd Reilly N . Real-world overall survival and treatment patterns by PTEN status in metastatic castration-resistant prostate cancer. JCO Precis Oncol. (2024) 8:e2300562. doi: 10.1200/po.23.00562 38547419 PMC10994466

[109] Jamaspishvili T Patel P Bennett M Bearden J Bixby A Desai D . Assessing the impact of PTEN loss on outcomes in high-/very high-risk, advanced hormone-sensitive prostate cancer patients: Institutional retrospective study. J Clin Oncol. (2025) 43:260. doi: 10.1200/jco.2025.43.5_suppl.260

[110] Lotan TL Gumuskaya B Rahimi H Hicks JL Iwata T Robinson BD . Cytoplasmic PTEN protein loss distinguishes intraductal carcinoma of the prostate from high-grade prostatic intraepithelial neoplasia. Mod Pathol. (2013) 26:587–603. doi: 10.1038/modpathol.2012.201 23222491 PMC3610824

[111] Morais CL Han JS Gordetsky J Nagar MS Anderson AE Lee S . Utility of PTEN and ERG immunostaining for distinguishing high-grade PIN from intraductal carcinoma of the prostate on needle biopsy. Am J Surg Pathol. (2015) 39:169–78. doi: 10.1097/pas.0000000000000348 25517949 PMC4293206

[112] Grünwald V DeGraffenried L Russel D Friedrichs WE Ray RB Hidalgo M . Inhibitors of mTOR reverse doxorubicin resistance conferred by PTEN status in prostate cancer cells. Cancer Res. (2002) 62:6141–5. 12414639

[113] Mulholland DJ Tran LM Li Y Cai H Morim A Wang S . Cell autonomous role of PTEN in regulating castration-resistant prostate cancer growth. Cancer Cell. (2011) 19:792–804. doi: 10.1016/j.ccr.2011.05.006 21620777 PMC3157296

[114] Carver BS Chapinski C Wongvipat J Hieronymus H Chen Y Chandarlapaty S . Reciprocal feedback regulation of PI3K and androgen receptor signaling in PTEN-deficient prostate cancer. Cancer Cell. (2011) 19:575–86. doi: 10.1016/j.ccr.2011.04.008 21575859 PMC3142785

[115] Shorning BY Dass MS Smalley MJ Pearson HB . The PI3K-AKT-mTOR pathway and prostate cancer: At the crossroads of AR, MAPK, and WNT signaling. Int J Mol Sci. (2020) 21:4507. doi: 10.3390/ijms21124507 32630372 PMC7350257

[116] Vidotto T Melo CM Castelli E Koti M Dos Reis RB Squire JA . Emerging role of PTEN loss in evasion of the immune response to tumours. Br J Cancer. (2020) 122:1732–43. doi: 10.1038/s41416-020-0834-6 32327707 PMC7283470

[117] Georgescu MM . PTEN tumor suppressor network in PI3K-Akt pathway control. Genes Cancer. (2010) 1:1170–7. doi: 10.1177/1947601911407325 21779440 PMC3092286

[118] Augello MA Liu D Deonarine LD Robinson BD Huang D Stelloo S . CHD1 loss alters AR binding at lineage-specific enhancers and modulates distinct transcriptional programs to drive prostate tumorigenesis. Cancer Cell. (2019) 35:603–17:e8. doi: 10.1016/j.ccell.2019.03.001 30930119 PMC6467783

[119] Toso A Revandkar A Di Mitri D Guccini I Proietti M Sarti M . Enhancing chemotherapy efficacy in Pten-deficient prostate tumors by activating the senescence-associated antitumor immunity. Cell Rep. (2014) 9:75–89. doi: 10.1016/j.celrep.2014.08.044 25263564

[120] Vidotto T Saggioro FP Jamaspishvili T Chesca DL Picanco de Albuquerque CG Reis RB . PTEN-deficient prostate cancer is associated with an immunosuppressive tumor microenvironment mediated by increased expression of IDO1 and infiltrating FoxP3+ T regulatory cells. Prostate. (2019) 79:969–79. doi: 10.1002/pros.23808 30999388

[121] Di Mitri D Mirenda M Vasilevska J Calcinotto A Delaleu N Revandkar A . Re-education of tumor-associated macrophages by CXCR2 blockade drives senescence and tumor inhibition in advanced prostate cancer. Cell Rep. (2019) 28:2156–68:e5. doi: 10.1016/j.celrep.2019.07.068 31433989 PMC6715643

[122] Maxwell KN Cheng HH Powers J Gulati R Ledet EM Morrison C . Inherited TP53 variants and risk of prostate cancer. Eur Urol. (2022) 81:243–50. doi: 10.1016/j.eururo.2021.10.036 34863587 PMC8891030

[123] Bian Z Chen J Wang Y Jin C Liang C Meng J . Modulating the immunosuppressive tumor microenvironment and inhibiting growth in Mutp53-driven CRPC via STAT3 pathway blockade. Int J Biol Sci. (2025) 21:3081–98. doi: 10.7150/ijbs.111732 40384867 PMC12080385

[124] Textor S Fiegler N Arnold A Porgador A Hofmann TG Cerwenka A . Human NK cells are alerted to induction of p53 in cancer cells by upregulation of the NKG2D ligands ULBP1 and ULBP2. Cancer Res. (2011) 71:5998–6009. doi: 10.1158/0008-5472.can-10-3211 21764762

[125] Veneziani I Infante P Ferretti E Melaiu O Battistelli C Lucarini V . Nutlin-3a enhances natural killer cell-mediated killing of neuroblastoma by restoring p53-dependent expression of ligands for NKG2D and DNAM-1 receptors. Cancer Immunol Res. (2021) 9:170–83. doi: 10.1158/2326-6066.cir-20-0313 33303573

[126] Nakano K Vousden KH . PUMA, a novel proapoptotic gene, is induced by p53. Mol Cell. (2001) 7:683–94. doi: 10.1016/s1097-2765(01)00214-3 11463392

[127] Guo G Yu M Xiao W Celis E Cui Y . Local activation of p53 in the tumor microenvironment overcomes immune suppression and enhances antitumor immunity. Cancer Res. (2017) 77:2292–305. doi: 10.1158/0008-5472.can-16-2832 28280037 PMC5465961

[128] Chipuk JE Kuwana T Bouchier-Hayes L Droin NM Newmeyer DD Schuler M . Direct activation of Bax by p53 mediates mitochondrial membrane permeabilization and apoptosis. Science. (2004) 303:1010–4. doi: 10.1126/science.1092734 14963330

[129] Izquierdo E Vorholt D Blakemore S Sackey B Nolte JL Barbarino V . Extracellular vesicles and PD-L1 suppress macrophages, inducing therapy resistance in TP53-deficient B-cell malignancies. Blood. (2022) 139:3617–29. doi: 10.1182/blood.2021014007 35344582

[130] Langenbach M Giesler S Richtsfeld S Costa-Pereira S Rindlisbacher L Wertheimer T . MDM2 inhibition enhances immune checkpoint inhibitor efficacy by increasing IL15 and MHC class II production. Mol Cancer Res. (2023) 21:849–64. doi: 10.1158/1541-7786.mcr-22-0898 37071397 PMC10524444

[131] Winter L Pawlowsky L Marcinek A Brauchle B Muth A Kazerani M . The battle within: AML´s p53 strategies to evade T-cell attack. Blood. (2023) 142:1411. doi: 10.1182/blood-2023-173826

[132] Wen GM Zhao ZY Zheng XH Liu DH Xia P . Effect of TP53 mutation, expression and polymorphism on the survival, immune infiltration and ferroptosis in patients with prostate cancer. Oncol Lett. (2025) 30:445. doi: 10.3892/ol.2025.15191 40740989 PMC12308458

[133] Dallos MC Obradovic AZ McCann P Chowdhury N Pratapa A Aggen DH . Androgen deprivation therapy drives a distinct immune phenotype in localized prostate cancer. Clin Cancer Res. (2024) 30:5218–30. doi: 10.1158/1078-0432.ccr-24-0060 39269310 PMC11905119

[134] Kalina JL Neilson DS Comber AP Rauw JM Alexander AS Vergidis J . Immune modulation by androgen deprivation and radiation therapy: Implications for prostate cancer immunotherapy. Cancers (Basel). (2017) 9:13. doi: 10.3390/cancers9020013 28134800 PMC5332936

[135] Sha K Zhang R Maolake A Singh S Chatta G Eng KH . Androgen deprivation triggers a cytokine signaling switch to induce immune suppression and prostate cancer recurrence. In: Biorxiv (2025). p. 2023.12.01.569685.

[136] Cifuentes-Rojas C Hernandez AJ Sarma K Lee JT . Regulatory interactions between RNA and polycomb repressive complex 2. Mol Cell. (2014) 55:171–85. doi: 10.1016/j.molcel.2014.05.009 24882207 PMC4107928

[137] Burr ML Sparbier CE Chan KL Chan YC Kersbergen A Lam EYN . An evolutionarily conserved function of polycomb silences the MHC class I antigen presentation pathway and enables immune evasion in cancer. Cancer Cell. (2019) 36:385–97:e8. doi: 10.1016/j.ccell.2019.08.008 31564637 PMC6876280

[138] Morel KL Sheahan AV Burkhart DL Baca SC Boufaied N Liu Y . EZH2 inhibition activates a dsRNA-STING-interferon stress axis that potentiates response to PD-1 checkpoint blockade in prostate cancer. Nat Cancer. (2021) 2:444–56. doi: 10.1038/s43018-021-00185-w 33899001 PMC8061902

[139] Fischetti I Botti L Sulsenti R Cancila V Enriquez C Ferri R . Combined therapy targeting AR and EZH2 curbs castration-resistant prostate cancer enhancing anti-tumor T-cell response. Epigenomics. (2024) 16:653–70. doi: 10.2217/epi-2023-0374 38530086 PMC11160446

[140] Guo B Tan X Cen H . EZH2 is a negative prognostic biomarker associated with immunosuppression in hepatocellular carcinoma. PloS One. (2020) 15:e0242191. doi: 10.1371/journal.pone.0242191 33180829 PMC7660515

[141] Wang L Jin Q Lee JE Su IH Ge K . Histone H3K27 methyltransferase Ezh2 represses Wnt genes to facilitate adipogenesis. Proc Natl Acad Sci USA. (2010) 107:7317–22. doi: 10.1073/pnas.1000031107 20368440 PMC2867706

[142] Li M Melnick AM . An "EZ" epigenetic road to leukemia stem cell metabolic reprogramming? Cancer Discov. (2019) 9:1158–60. doi: 10.1158/2159-8290.cd-19-0737 31481404 PMC10594541

[143] Li H Gigi L Zhao D . CHD1, a multifaceted epigenetic remodeler in prostate cancer. Front Oncol. (2023) 13:1123362. doi: 10.3389/fonc.2023.1123362 36776288 PMC9909554

[144] Shenoy TR Boysen G Wang MY Xu QZ Guo W Koh FM . CHD1 loss sensitizes prostate cancer to DNA damaging therapy by promoting error-prone double-strand break repair. Ann Oncol. (2017) 28:1495–507. doi: 10.1093/annonc/mdx165 28383660 PMC5834074

[145] Zhang Z Zhou C Li X Barnes SD Deng S Hoover E . Loss of CHD1 promotes heterogeneous mechanisms of resistance to AR-targeted therapy via chromatin dysregulation. Cancer Cell. (2020) 37:584–98:e11. doi: 10.1016/j.ccell.2020.03.001 32220301 PMC7292228

[146] Zhao D Lu X Wang G Lan Z Liao W Li J . Synthetic essentiality of chromatin remodelling factor CHD1 in PTEN-deficient cancer. Nature. (2017) 542:484–8. doi: 10.1038/nature21357 28166537 PMC5448706

[147] Cheng B Peng W . Mp32-03 Arid1a-mediated phase separation drives immune evasion and impairs immunotherapy in prostate cancer. J Urol. (2025) 213. doi: 10.1097/01.ju.0001110112.97722.d8.0333079766

[148] Greene S Robbins Y Mydlarz WK Huynh AP Schmitt NC Friedman J . Inhibition of MDSC trafficking with SX-682, a CXCR1/2 inhibitor, enhances NK-cell immunotherapy in head and neck cancer models. Clin Cancer Res. (2020) 26:1420–31. doi: 10.1158/1078-0432.ccr-19-2625 31848188 PMC7073293

[149] Kloss CC Lee J Zhang A Chen F Melenhorst JJ Lacey SF . Dominant-negative TGF-beta receptor enhances PSMA-targeted human CAR T cell proliferation and augments prostate cancer eradication. Mol Ther. (2018) 26:1855–66. doi: 10.1016/j.ymthe.2018.05.003 29807781 PMC6037129

[150] Lyu A Fan Z Clark M Lea A Luong D Setayesh A . Evolution of myeloid-mediated immunotherapy resistance in prostate cancer. Nature. (2025) 637:1207–17. doi: 10.1038/s41586-024-08290-3 PMC11779626 39633050

[151] Hailemichael Y Johnson DH Abdel-Wahab N Foo WC Bentebibel SE Daher M . Interleukin-6 blockade abrogates immunotherapy toxicity and promotes tumor immunity. Cancer Cell. (2022) 40:509–23:e6. doi: 10.1016/j.ccell.2022.04.004 35537412 PMC9221568

[152] Schweizer MT Calvo M Moreno V Mellado B Castellano D Spira AI . Mevrometostat (PF-06821497), an enhancer of zeste homolog 2 (EZH2) inhibitor, in combination with enzalutamide in patients with metastatic castration-resistant prostate cancer (mCRPC): A randomized dose-expansion study. J Clin Oncol. (2025) 43:LBA138–LBA. doi: 10.1200/jco.2025.43.5_suppl.lba13842852411

[153] Barnett RM Jang A Lanka S Fu P Bucheit LA Babiker H . Blood-based tumor mutational burden impacts clinical outcomes of immune checkpoint inhibitor treated breast and prostate cancers. Commun Med (Lond). (2024) 4:256. doi: 10.1038/s43856-024-00687-5 39623081 PMC11612475

[154] Wu K Liang J Shao Y Xiong S Feng S Li X . Evaluation of the efficacy of PARP inhibitors in metastatic castration-resistant prostate cancer: A systematic review and meta-analysis. Front Pharmacol. (2021) 12:777663. doi: 10.3389/fphar.2021.777663 34975480 PMC8718674

[155] Zhou T Li Y Liu Z Zeng Z Li T Chen L . IL-6/STAT3 signaling in prostate cancer: CAF-driven immune evasion and therapeutic opportunities. Front Immunol. (2026) 16:1736606. doi: 10.3389/fimmu.2025.1736606 PMC12873542 41660621

[156] Qin Z Liu R Jing S Guo C Li W . Immune-inflammation index as prognostic markers in metastatic castration-resistant prostate cancer: a systematic review and meta-analysis. Front Oncol. (2026) 16:1806929. doi: 10.3389/fonc.2026.1806929 42125686 PMC13158060

[157] Amori G Sugawara E Shigematsu Y Akiya M Kunieda J Yuasa T . Tumor B7-H3 expression in diagnostic biopsy specimens and survival in patients with metastatic prostate cancer. Prostate Cancer Prostatic Dis. (2021) 24:767–74. doi: 10.1038/s41391-021-00331-6 33558663

[158] Ju Y Watson J Wang JJ Yen YT Gevorkian L Chen Z . B7-H3-liquid biopsy for the characterization and monitoring of the dynamic biology of prostate cancer. Drug Resist Update. (2025) 79:101207. doi: 10.1016/j.drup.2025.101207 39914189 PMC12705526

[159] Petrylak DP Ratta R Matsubara N Korbenfeld E Gafanov R Mourey L . Pembrolizumab plus docetaxel versus docetaxel for previously treated metastatic castration-resistant prostate cancer: The randomized, double-blind, phase III KEYNOTE-921 trial. J Clin Oncol. (2025) 43:1638–49. doi: 10.1200/jco-24-01283 40043230 PMC12058370

[160] Graff JN Burotto M Fong PC Pook DW Zurawski B Manneh Kopp R . Pembrolizumab plus enzalutamide versus placebo plus enzalutamide for chemotherapy-naive metastatic castration-resistant prostate cancer: the randomized, double-blind, phase III KEYNOTE-641 study. Ann Oncol. (2025) 36:976–87. doi: 10.1016/j.annonc.2025.05.007 40383193

[161] Gratzke C Ozguroglu M Peer A Sendur MAN Retz M Goh JC . Pembrolizumab plus enzalutamide and androgen deprivation therapy versus placebo plus enzalutamide and androgen deprivation therapy for metastatic hormone-sensitive prostate cancer: the randomized, double-blind, phase III KEYNOTE-991 study. Ann Oncol. (2025) 36:964–75. doi: 10.1016/j.annonc.2025.05.008 40383194

[162] Fizazi K Saad F Alonso-Gordoa T Zurawski B Barthelemy P Voog E . Nivolumab plus docetaxel versus placebo plus docetaxel for androgen receptor pathway inhibitor-pretreated and chemotherapy-naive metastatic castration-resistant prostate cancer (CheckMate 7DX): a double-blind, randomised, phase 3 trial. Lancet Oncol. (2026) 27:68–78. doi: 10.1016/s1470-2045(25)00566-2 41449150

[163] Sharma P Krainer M Saad F Castellano D Bedke J Kwiatkowski M . Nivolumab plus ipilimumab for chemotherapy-refractory metastatic castration-resistant prostate cancer: results from the randomized portion of the phase 2 CheckMate 650 trial. Nat Commun. (2026) 17:6216. doi: 10.1038/s41467-026-72242-w 42098105 PMC13369907

[164] Dorff TB Blanchard MS Adkins LN Luebbert L Leggett N Shishido SN . PSCA-CAR T cell therapy in metastatic castration-resistant prostate cancer: a phase 1 trial. Nat Med. (2024) 30:1636–44. doi: 10.1038/s41591-024-02979-8 38867077 PMC11186768

[165] Aggarwal R Rottey S Bernard-Tessier A Mellado B Kosaka T Stadler WM . Safety and efficacy of Tarlatamab in patients with neuroendocrine prostate cancer: Results from the phase 1b DeLLpro-300 study. Clin Cancer Res. (2025) 31:3854–63. doi: 10.1158/1078-0432.ccr-25-1211 40689871 PMC12434391

[166] Canale FP Basso C Antonini G Perotti M Li N Sokolovska A . Metabolic modulation of tumours with engineered bacteria for immunotherapy. Nature. (2021) 598:662–6. doi: 10.1038/s41586-021-04003-2 34616044

